# Effects of spinal deformities on lower limb kinematics during walking: a systematic review and meta-analysis

**DOI:** 10.1038/s41598-025-88886-5

**Published:** 2025-02-07

**Authors:** Fateme Khorramroo, Seyed Hamed Mousavi, Reza Rajabi

**Affiliations:** Department of Sport Injuries and Biomechanics, Faculty of Sport Sciences and Health, University of Tehran, Tehran, Iran

**Keywords:** Malalignment, Biomechanics, Scoliosis, Kyphosis, Lordosis, Spatiotemporal, Gait, Rehabilitation, Disability

## Abstract

Spinal deformities characterized by malalignment of the spinal column, including scoliosis, hyper kyphosis, and lordosis, can significantly impact lower limb kinematics, potentially leading to altered gait patterns. We aimed to synthesize existing literature investigating the effect of spinal deformities on lower limb kinematics during walking. We searched Four databases including PubMed, Web of Science, Scopus and Embase from their inception through 29th December 2024. The Newcastle-Ottawa Scale was applied to assess quality of included studies and the Grading of Recommendations Assessment, Development and Evaluation (GRADE system) was employed to assess the overall quality of the evidence in the meta-analysis. Mean differences and 95% confidence intervals (CI) were calculated with random effects model in RevMan version 5.4. The review included 35 studies with 1941 participants. Regarding scoliosis, strong evidence suggested a significant increase in right leg stance% and non-significant changes in left leg cadence, right and left leg speed and ankle transverse ROM in individuals with scoliosis compared to controls. Sensitivity analysis showed strong evidence of decrease in right leg stride length and ankle sagittal ROM, decrease in left leg single support and non-significant change in left leg step length. Individuals with sagittal malalignment showed strong evidence of significant reduction in velocity; moderate evidence suggested a significant increase in double support and a decrease in stride length, knee and hip sagittal ROM. Sensitivity analysis revealed strong evidence of a significant decrease in cadence during walking in individuals with sagittal trunk malalignment compared to controls. Participants with adult spinal deformity exhibited strong evidence of increased double support and decreased cadence during walking compared to controls. Spinal deformities significantly alter lower limb kinematics during gait. Our findings highlight the essential need for clinicians to take gait alterations into account when developing targeted interventions for individuals with spinal deformity.

**Supplementary Information:**

The online version contains supplementary material available at 10.1038/s41598-025-88886-5.

## Introduction

Spinal deformities are characterized by abnormal curvature and alignment of the spine^1,2^. The common forms of spinal deformities in frontal plane include scoliosis and in sagittal plane includes hyper kyphosis, lordosis, as well as flatback and swayback syndromes^3^. Adult scoliosis is defined as a spinal deformity in skeletally mature patients with a coronal Cobb angle greater than 10°, which can be idiopathic or degenerative due to age-related changes like disc degeneration and vertebral fractures^4^. Adolescent idiopathic scoliosis affects 1–4% of adolescents^5^. However, the overall prevalence was reported 0.47–5.2%. The ratio of females to males varies between 1.5:1 and 3:1, and this ratio significantly rises with age^6^. Adult spinal deformity (ASD) includes various three-dimensional spinal abnormalities, such as scoliosis, hyper kyphosis, and sagittal imbalance^7^, affecting 2–32% of adults and up to 68% of the elderly^8^. Hyper kyphosis features excessive outward thoracic curvature, while lordosis involves an exaggerated inward lumbar curve. Spinal deformities leads to progressive changes that result in pain, neurological^9^ and respiratory symptoms^10^, and functional impairment, with manifestations ranging from cosmetic concerns to severe discomfort^9^ and hindering rehabilitation efforts^11^. These conditions profoundly influence biomechanics and gait^12,13^.

Scoliosis, adult spinal deformity (ASD), and sagittal malalignment profoundly influence lower limb gait kinematics and spatiotemporal parameters^14^. During locomotion, these spinal deformities can disrupt lower limb biomechanics through a kinematic chain and fascial slings^15^, leading to compensatory adjustments in contralateral side^16^. Changes such as altered cobb angle, pelvic tilt and trunk flexion can manifest as slower walking speed^17^, reduced stride lengths^16^, and increased joint stress^11^. However, it is noteworthy that some studies indicated no significant change in these parameters^18–20^. This disruption in normal biomechanics during alternating open and closed kinematics chain of walking, not only exacerbates back pain^12^ and muscle fatigue^21^ but also accelerates joint degeneration, ultimately restricting daily activities and diminishing overall health-related quality of life^22^. Understanding these interconnected changes is essential for developing effective management and rehabilitation strategies for individuals affected by these conditions.

While these studies contribute significantly to foundational knowledge, they often fail to integrate these insights into a cohesive framework. Moreover, two studies with similar titles systematically reviewed publications in this area^23,24^, they had different aims and inclusion criteria from our research. This lack of comprehensive synthesis highlights a significant gap in literature and underscores the need for quantitative insights into the impact of spinal deformities on gait kinematics. In light of this, the current study aims to bridge these gaps by systematically reviewing existing studies which assessed the impact of spinal deformities on lower limb kinematics (i.e., joint angles and spatiotemporal) during walking and to perform a meta-analysis to quantify the differences in gait parameters between individuals with spinal deformities and healthy controls. Understanding these differences in lower limb kinematics of individuals with spinal deformity will provide valuable insights for clinical decision making, developing targeted rehabilitation strategies and ultimately improving the management of individuals with spinal deformities.

## Methods

This systematic review was executed following the PERSiST guidelines established for systematic reviews^25^ and registered the protocol in PROSPERO (*CRD42024592317*).

### Search strategy

We located pertinent studies using four electronic databases: PubMed, Web of Science, Scopus, and Embase. The search was conducted on 29th December 2024. The key terms employed in our search strategy included broad terms, divided into 3 specific categories:

#1 malalignment OR kyphosis OR lordosis OR “flat back” OR “sway back” OR “forward head” OR “upper cross syndrome” OR spinal or “spinal deformity” OR deformity OR scoliosis OR scoliotic.

#2 kinematic OR ankle OR knee OR hip OR tibia OR stance OR acceleration OR velocity OR displacement OR spatiotemporal OR speed OR cadence OR “step length” OR “stride length” OR “step width” OR “stride time” OR “step rate” OR “double support” OR “single support” OR inversion OR eversion OR dorsiflexion OR plantarflexion OR pronation OR supination.

#3 walk.

#4 (1 AND 2 AND 3).

Additionally, we manually searched reference lists of previous studies, related systematic reviews and google scholar on association of spinal deformities with lower limb kinematics during gait to ensure comprehensive identification of all applicable studies.

### Eligibility criteria

Independent searches were performed based on predefined inclusion criteria and data extraction forms. Included studies were limited to those published in English and examined lower limb kinematics in both groups. Studies were excluded if they were non-English, focused solely on individuals without spinal deformities, did not report on lower limb kinematics, lacked a control group of healthy participants, concentrated on spinal injuries or post-operative changes, or were categorized as e-posters, conference abstracts, or unavailable research papers.

### Study selection

The review process based on inclusion criteria for titles, abstracts, and full texts was conducted by (FK and SHM) in accordance with the established inclusion criteria. In cases of disagreement, the two authors discussed the manuscript to achieve a consensus.

### Quality assessment

The risk of bias of the included non-randomized trials was evaluated by FK and SHM using the Newcastle-Ottawa Scale, specifically adapted for cross-sectional studies (scores ≥ 7–9, 4–6, < 4 are considered poor, fair, and high, respectively)^26^. However, since the responsiveness question is not applicable to our studies, the total score was determined to be 8. The Grading of Recommendations Assessment, Development and Evaluation (GRADE system)^1^ was employed to assess the overall quality of the evidence in the meta-analysis.

### Data collection

Data extraction from the included studies was performed by (FK), with verification completed by (SHM). For consistency, data were categorized by type of spinal deformity in the Results and Discussion sections. Extracted variables included inclusion criteria, type of deformity, study design, participant characteristics (age, sex, height, mass, BMI), tools, source of fund and tools utilized.

### Synthesis of results

Mean differences or standardized mean differences along with 95% confidence intervals (CIs) were calculated using a random effects model in RevMan version 5.4. A meta-analysis was performed when at least two studies assessed the same outcome measure using similar methodologies. The statistical heterogeneity of the pooled data was evaluated through I² statistics and their associated P-values (*P* < 0.05). To assess the impact of each study and the robustness of the final results, a sensitivity analysis^27^ was performed by sequentially excluding individual studies from the analysis. The results were interpreted according to the levels of evidence as outlined by van Tulder et al.^28^ modified by Mousavi et al.^29^ (Table 1).

**Table 1:** Definitions of modified level of evidence.

Level of evidence	Description
Strong evidence	Pooled results from three or more studies, including a minimum of two high-quality studies which are statistically homogenous (*p*>0.05)- may be associated with a statistically significant or non-significant pooled results.
Moderate evidence	Statistically significant pooled results from multiple studies, including at least one high-quality study, which are statistically heterogeneous (*p*<0.05); or from multiple low- or moderate-quality studies which are statistically homogenous (*p*>0.05); or statistically insignificant pooled results from multiple studies, including at least one high-quality study, which are statistically homogenous (*p*>0.05).
Limited evidence	Results from multiple low- or moderate-quality studies which are statistically heterogeneous (*p*<0.05); or from one high-quality study.
Very limited evidence	Results from one low- or moderate-quality study.
Conflicting evidence	Pooled results that are insignificant and from multiple studies, regardless of quality, which are statistically heterogeneous (*p*<0.05, i.e., inconsistent).

## Results

### Study selection

**Fig. 1:**
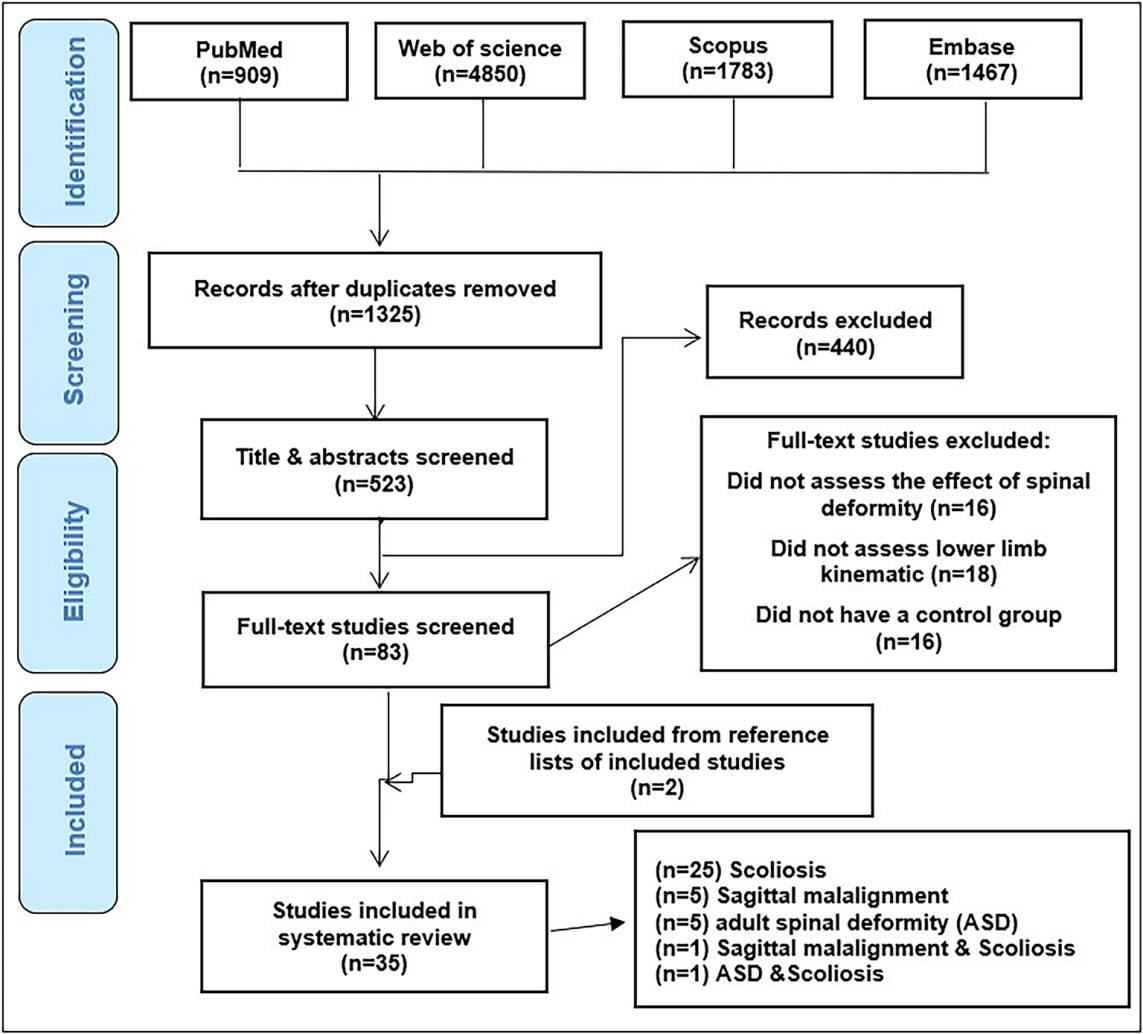
Flow chart of study selection process

The main literature search yielded a total of 9009 items from which 1325 items remained after duplicate removal: PubMed (909 studies), Web of Science (4850), Scopus (1783) and Embase (1467). We excluded 1292 studies due to not meeting the inclusion criteria and included 33 studies after screening of titles, abstracts and full text for further eligibility check. Two studies added by hand search. A total of 35 studies were included. Figure 1. shows the flow diagram of the selection process and number of excluded studies at each stage.

### Study characteristics

Table 2. summarizes the characteristics of the included studies. Twenty five studies examined the impact of scoliosis, six studies investigated the effects of sagittal malalignment, and three studies analyzed the influence of ASD on lower limb kinematics. Additionally, one study compared both scoliosis and sagittal malalignment, while another study assessed the differences between ASD and scoliosis in relation to healthy controls. Total sample size of included studies was 1941.

**Table 2:** Study characteristics.

Authors	Type	Measured kinematics outcomes	spinal angles or inclusion	Sample size	Study design	Age	Height (cm)	Weight (kg)	BMI (kg/m 2)	Measurement tools	Funding and support
**Park et al. 2015**	Adolescent Idiopathic Scoliosis	Speed, stride length, stance phase	Cobb angle > 10, 23 patient main curve at thoracic and 16 at thoracolumbar level	E: 39, C: 30	Case-control	E: 15.1 ± 2.1, C: 14 8 ± 2.7	E: 155.2 ± 8.2, C: 154.9 ± 5.6	E: 45.6 ± 9.5, C: 44.7 ± 6.3		6 camera 460, Vicon motion system, Oxford, UK	
**Haber et al. 2015**	Scoliosis	Speed, stride time, length	Scoliometric reading of at least 5°, Leg length discrepancy (LLD) < 2 cm	E: 31, C: 31 M a&F	Case-control	10 to 30				6 video cameras VICON	Salvatore Maugeri Research Award
**Yang et al. 2013**	Idiopathic Scoliosis	Joint angles, velocity, cadence, stride length, step length, and the relative and absolute durations of the individual gait phases: total stance time, single-limb stance time and dou-ble limb stance time for each side.	cob angle (lumbar: 0-18, Thoracolumbar: 0-31, Thoracic: 0-34), right thoracic and left lumbar scoliosis, the left convex on lumbar, right convex on thoracic spine	E: 2 M, 18 F, C: 5 M, 15 F	Case-control	E: 14.9 ± 1.0, C 14.4 ± 1.0	E: 161.6 ± 6.6, C: 160.9 ± 5.7	E: 59.2 ± 7.6, C: M53.3 ± 9.8		6 digital cameras (Motion Analysis Corporation, Santa Rosa, CA, USA)	Supported by the Institute of Health Science Grant at Korea University and the Basic Science Research Program through the National Research Foundation of Korea funded by the Ministry of Education, Science and Technology (2010-0003015). Part of this study was supported by the Korea Healthcare Technology R&D project (A110416).
**Mahaudens et al. 2005**	Idiopathic Scoliosis	Speed, step length, cadence, stance phase%	X-ray evidence of progressive idiopathic scoliosis with a lumbar or thoraco-lumbar curve	E: 12, C: 12	Case-control	E: 13.2±0.8, C: 12.9±0.9	E: 1.56±0.11, C: 1.58±0.11	E: 41.2±0.8, C: 46.4±9		4 infrared cameras, EMG	none
**Yazdani et al. 2015**	Idiopathic Scoliosis	Spatiotemporal variables including velocity, cadence, stride and step length, initial heel contact and toe off instants (% of gait cycle), duration of single and double supports, duration of the steps and strides	Cobb angle > 10°, Cobb angle:27.8±8.5, vertebral rotation: 1.94°±0.4, convex on the right thoracic side	E: 19 F, C: 18 F	Case-control	E: 14±1.3 C: 14.1±1.5	E: 163.7±5.4, C:161.2±5.23	P; 49±8, C: 49.6±7.7	E: 18.6±2.4, C: 19.3±2.3	4cameras، VICON 460	None
**Lao et al. 2001**	Adolescent Idiopathic Scoliosis	Spatiotemporal	Cobb angle: 10°-45°	E: 18 F, C: 8 F	Case-control	E: 14.3 ± 1.4, C: 14.8 ± 1.3				Vicon 370	None
**Wu et al. 2019**	Adolescent Idiopathic Scoliosis	Spatiotemporal, (hip, knee, ankle) Angles	Lenke 1: Cobb angles 52.7° ± 10.5°	E: 16 F, C: 16 F	Case-control	14.9 ± 1.7	154.7 ± 5.0 cm	41.7 ± 7.2		8-camera, Vicon 120 Hz	Financial support (NSC98-2320-B-039-041) by National Science Council, Taiwan
**Chen et al. 1998**	Adolescent Idiopathic Scoliosis	Cadence, stride length	Cobb Angle: 22°-67°	p; 2 M, 28 F c:2 M, 13 F	Case-control	E: 16.6±3.8, c: 16.8±3.1			.	8 video cameras 100 Hz	Support was provided by the National Science Council, NSC-85-2331-B-002-128, Taiwan, and Taiwan Spine Research Foundation.
**Mahaudens et al. 2009**	Adolescent Idiopathic Scoliosis	angular displacement and angular speed of hip, knee, and ankle, step length, step	group 1 (Cb **≤** 20°), group 2 (20 ˂ Cb ˂ 40°) and group 3 (Cb ≥ 40°). A left thoracolumbar or lumbar primary structural curve (types 5 and 6)	E: 41 F, C: 13 F	Case-control	E: mean: 16, c: mean:16	E: 163.4 ±8.5, c: 164.3 ±4	E: 5 0.2±7.1, c: 54.7 ±2.92.9	E: 18.8±2.1, c: 20.2±1	4 infrared cameras measured at 100 Hz	None
**Tekin et al. 2023**	Adolescent Idiopathic Scoliosis	Step time/length, single/double support time, cadence, foot progression angle, cadence, velocity, base of support	Single or double curve pattern	E: 7 M, 19 F C: 8 M, 13 F	Case-control	E: 14.9 ± 2.6, C : 14.5 ± 3.2	E: 163.6±11.2, C:156.7±19.7	E: 51.9 ± 11.4, C:50.7 ± 19.1	E: 19.2±2.9, C: 19.7±3.6	smartphone camera, Kinovea Software	None
**Haddas et al. 2018**	Adult degenerative scoliosis	Gait parameter, knee flexion, knee ROM	Cobb Angle > 25°	E: 20, C: 15	Case-control	E: 62.71 ± 6.9, C: 55.01±6.1	E: 164±0.1, C:171±0.1	E: 79.37 ± 22.2, C:72.14 ± 15.7		10 cameras, Vicon Nexus 2.0 Inc.	None
**Engsberg eta al. 2003**	Lumbar scoliosis	Spatiotemporal, Knee, Hip flexion	Fixed and not fixed sagittal imbalance	E: 8 F, C: 9 F	Case-control					6 camera HiRes Motion 6 Analysis Cor- poration System 60 Hz	None
**Alazzawi et al. 2023**	Adolescent Idiopathic Scoliosis	Spatiotemporal	less than 20 degrees, to even more than 45 degrees of Cobb’s angle	E: 20, c: 20M &F	Case-control		E:1.64 ±0.1, c: 1.71±0.1	E:79.37±22.2, c:72.14±15.7		Biodex Unweighting system	None
**Bortone et al. 2020**	Adolescent Idiopathic Scoliosis	gait speed, cadence, step length, hip, knee and ankle ROM	Thoracic or lumbar curve or thoracolumbar curve	E: 12 F, C: 12 F	single senter prospective study	13.8 ± 2.2 (12 - 17)	153.1 ± 6.6 (142 - 171)	48.9 ± 4.2 (45 - 59)	20.9 ± 1.9 (16.9 - 23.1)	8 Camera motion analysis system (BTS SMART DX6000, BTS SpA, Italy) at 240 Hz	None
**Schmid et al. 2016**	Adolescent Idiopathic Scoliosis	Spatiotemporal	both a thoracic and a thoracolumbar/lumbar curve component	E: 2 M, 12 F C: 8 M, 7 F	Cross sectional observational study	E: 15.2, c: 1.62	E: 152, c: 162	p; 55.6, c: 54.2		12 Vicon 200- 300 Hz	None
**Delpierre et al. 2019**	Idiopathic Scoliosis	Spatiotemporal	E: cobb angle: 56	E: 4 M, 18 F C: 4 M, 18 F	retrospective study	E: 16.9±3.1, C: 18.5±0.8	E: 162.4±8.4, C: 169.5±8.1	E: 52.1±8.4, C: 60.8±9.6		Vicon motion capture and Nexus software	None
**Huysmans et al. 2024**	Idiopatic and de novo scoliosis	Spatiotemporal, sagittal ROM of hip, knee, ankle	Indication for spinal fusion, idiopatic or de novo scoliosis	E: 24, C: 50 M &F	observational retrospective case-control	E: 20, C: 22	E: 171 C: 173	E: 68.5, c: 70.3		12 cameras, Vicon 100 Hz	None
**Mar et al. 2020**	ASD	Cadence, speed, stride length and step width	PI-LL>10, SVA>40, PT>20, coronal cobb angle 38-40	E: 40 F, 12 M, C: 25 F, 21 M	Retrospective	E: 58.1 ±15.8, 43 ±14	E: 1.6 ± 0.1, C: 1.7 ± 0.1	E: 71 ± 18.6, C: 73.9 ± 15.8	BMI: 26.8 ± 5.1, C: 73.9 ±15.8	3D, Vicon, Oxford, UK	None
**yagi et al. 2016**	ASD ( scoliosis and kyphosis and pelvic tilt)	Spatiotemporal	main-curve Cobb angle > 20° or a C7 sagittal vertical axis (SVA) > 5 cm	E: 33 F, C: 33 F	Prospective case series	E: 22.8 ± 2.5, C: 23.9±2.5	E: 150.9±7.9, C: 148.9±04.7	E: 52.5±2.5, C: 53.3±7.3	E: 22.8±2.5, C: 23.9±2.5		None
**Rebeyrat et al. 2022**	ASD ( scoliosis and kyphosis and pelvic retroversion)	Spatiotemporal	pelvic tilt (PT) > 25°, frontal Cobb angle > 20°, sagittal vertical axis (SVA) > 50 mm, or thoracic kyphosis (TK) > 60°	E:69, C: 62	Prospective case series	E: 51 ±20, c: 34 ±13				7 cameras, Vicon	Funded by the University of Saint-Joseph (grant FM361), EUROSPINE (TFR2020#22), and ParisTech BiomecAM chair program on subject-specifc musculoskeletal modeling (with the support of ParisTech and Yves Cotrel Foundations, Société Générale, Proteor and Covea). The funding sources did not intervene in study design; in the collection, analysis, and interpretation of data; in the writing of the report; and in the decision to submit the article for publication
**Kawkabani et al. 2021**	ASD ( scoliosis and kyphosis and pelvic tilt)	Spatiotemporal, hip and knee joints kinematics	PT > 25 ◦, SVA > 50 mm, Cobb angle > 20 ◦, and/or TK > 60 ◦	E:13 M, 39 F C: 31 M, 32 F	retrospective study	E: 43 ±21, c: 40 ±12	E: 163 ±11, c: 167 ±9	E: 71 ±16, c: 72 ±14		8 cameras, Vicon 200 Hz	Funded by the University of Saint-Joseph (grant FM361) and EUROSPINE (TFR2020#22). The funding sources did not intervene in study design; in the collection, analysis and interpretation of data; in the writing of the report; and in the decision to submit the article for publication.
**Severijn et al. 2021**	Scolios, kyphos, pelvic tilt	Spatiotemporal, Hip, ankle and knee joints kinematics	ASD 1: (SVA ≥ 4 cm with PI-LL>10˚ and/or PT>20˚) ± coronal deformity, ASD 2: (SVA ≤ 4 cm with PI-LL>10˚ and/or PT>20˚) ± coronal deformity, ASD 3: (Cobb angle ≥ 20˚)	E:43, C: 18M &F	Prospective study.	E: 62±14.3, c: 64.5±15	E:167.5±7.8, c: 160.3±9	E: 66.9±14.1, c: 63.2±13.2	E:23.9±3.4, c: 24.5±5.3	10 cameras, Vicon 100 Hz	KU Leuven C2 funds, Medtronic and a strategic basic research PhD grant (SB/ 1S56017N) of the Research Foundation − Flanders (FWO).
**Semaan et al. 2022**	ASD ( scoliosis and kyphosis and pelvic retroversion)	Hip, Knee and Ankle joints kinematics	PT > 25°, SVA > 50 mm, PI-LL > 10°, Cobb angle > 20° and/or T1T12 > 60°	E: 119, C: 60	cross-sectional	E: 51.5±19.2, c: 48.6±10.1	E:161.9±9.5, c: 166.1±8	E: 72.1±14.5, c: ±12.7		8 cameras, Vicon 200 Hz	None
**Nassim et al. 2024**	ASD	Hip and knee angles and spatiotemporal	PT > 25◦, SVA>50 mm, Cobb angle>20◦, PI-LL>10◦ and/or T1T12 thoracic kyphosis TK > 60◦	E: 93 F, 31 M, C: 32 F, 15 M	Prospective study	E: 54±19, C: 53±8	E:161 ± 10, C: 165 ± 8	E: 72 ± 14, C: 73 ± 11		Eight infrared cameras (Vicon Motion SysteM	funded by the University of Saint-Joseph (grant FM361) and EUROSPINE (TFR 2020#22). The funding sources did not intervene
**Lewis et al. 2015**	Healthy mocking Sway back, forward flexed and normal	Kinematic variables, joint motion		15 M &F	Case-control	mean: 29.5	mean: 170.6	meaqn: 6823.2		6 camera, motion capture system (Motion Analysis Corp., Santa Rosa, CA, USA)	None
**Sinaki et al. 2005**	hyperkyphosis	Step and stride length, velocity, cadence, single support, step width	Osteoporosis and thoracic hyper kyphosis, sagittal cobb angle= 50-65	E: 12, C: 13	Case-control	E: 76.5 ± 5, C: 71 ± 5	158.8 ± 5, C: 161 ± 4	E: 61 ± 8, C: 66 ± 12		10-camera (motion analysis, Santa Rosa, California)	None
**Saha et al. 2008**	Trunk flexion	Spatiotemporal, hip, ankle and knee ROM		7 M, 7 F	Case-control	mean: 25.6 ±12.6	mean: 174.2±19.9	mean: 72.3±112.1		Eagle Digital RealTime motion capture system, 120 Hz.	Supported by the National Institute on Disability and Rehabilitation Research of the United States Department of Education under grant H133E030030. The opinions in this publication are those of the grantee and do not necessarily reflect those of the U.S. Department of Education.
**Sarwahi et al. 2002**	Flatback	Spatiotemporal, hip, ankle and knee ROM		E: 3 M, 18 F, C: 23	prospectiv study	E: mean: 53, c: mean: 45				six-camera videobased, passive marker gait analysis system (Motion Analysis Corp., Santa Rosa, CA)	None
**Igawa et al. 2022**	Dropped head	Spatiotemporal and ankle, knee and hip angles		E: 2 M, 10 F C: 2 M, 10 F	prospectiv study	E: 73.5±4.1, c: 72.5±3.8	E: 151.4±7.9, c:156.5±9.9	E: 46.9±5.6, c: 54.310.5		10 cameras, Vicon, visual 3D analytical software	None
Gariepy et al.2009	Adolescent Idiopathic Scoliosis	Spatiotemporal	Cobb angle: 56 (± 12)	e:15f c:15f	Comparative	e:12.7 (1.4), c: 10.5 (1.8)	e: 1.55 (0.08), c: 1.43 (0.13)	e: 54.32 (12.69), c: 39.01 (10.85)		two AMTI force plates (Newton, MA)	None
Mahaudens et al. 2014	Adolescent Idiopathic Scoliosis	Spatiotemporal and hip, knee and ankle angles	skeletal immaturity, Risser 0 to 2 premenarcheal or postmenarcheal by less than one year, and a 25–40° Cobb angle on posteroanterior view radiographs. Thoracolumbar/lumbar Cobb angle: 26.6(9.8) [15-40]°, only convex side reported	e: 13f, c: 13f	Clinical prospective	e: 14 [12-15],	e: 157±8,	e: 48.5±8.4	e: 19.5±1.9	EMG telemetry system (Telemg, BTS, Italy), strain gauges measuring 3D-ground reaction forces	none
Nadi et al. 2018	Scoliosis	Hip, knee and ankle angles	25-37 degree of curve	e: 5, c: 5	Comparative	Height age gender matched	e: 1.58±0.5, c:1.53.5±0.8	e: 41.2±1.2, c: 35.6±6.1		3D motion analysis system, with seven high speed cameras (Qualysis Motion analysis system). A Kistler force plate	None
Garg et al. 2021	Scoliosis	Spatiotemporal	Main thoracic: 78.62 (8.10), thoracolumbar/lumbar: 60.52 (7.42), Pelvic tilt: 21.2 (5.97)°, Pelvic incidence:46.33 (10.97)°, Sacral slope: 25.05 (12.07)°, Sagittal vertical axis: -19.75 (4.64) mm, Lumber lordosis: 46.32 (4.84)°, Thoracic kyphosis: 24.57 (3.56)°, C7 offset: 6.7 (3.3) mm, patients with C7: 15% (3/20)%	E: 6 m, 14f, C: 7 m, 13f	Prospective comarative	e:15.25 ± 3.48, c: 16.4 ±2.84	e: 148.13 (8.14), c: 151.60 (5.32)	e: 42.45 (8.24), c: 44.36 (8.27)		force plate (BTS, Italy)	Council of Scientific and Industrial Research provided research associate fellowship
Suh et al. 2023	Adolescent Idiopathic Scoliosis	Spatiotemporal	leg length discrepancy less than 1 cm	e: 59f, 9 m, c: 14f, 3 m	Observational comparison	e: 14.50 (6.98), c: 12.41 (2.21)	e: 1.59 (0.08), c: 1.53 (0.14)	e: 47.43 (6.98), c: 51.02 (12.77)	e: 18.73 (2.40), c: 21.56 (3.02)	2 force plates (Kistler, Type 5233 A, Switzerland)	None
Wu et al. 2020	Adolescent Idiopathic Scoliosis	Spatiotemporal	Scoliosis Lenke 1, hyperkyphosis	E: 16f, c: 16f	Cross-sectional	e: 14.0±1.8, c: 14.4±2.0	e: 154.8±4.7, c: 158.4 ±6.2	e: 42.0±7.5, c: 48.6±8.9		Three-dimensional 8-camera motion analysis system (Vicon MX T-40, OMG, U.K.) at 120 Hz	None

Notes: E; Experimental, C: Control, ASD; Adult spinal deformity, ROM; Range of motion, TK: Thoracic kyphosis, SVA: Sagittal vertical axis, PT: Pelvic tilt, PI-LL: difference between pelvic incidence and lumbar lordosis.

### Quality assessment

Table 3. shows the results of Newcastle-Ottawa Scale. The average score of eligible studies was 7.3 indicating high quality. The overall GRADE system quality of evidence was rated as high, high and moderate quality for scoliosis, ASD and Sagittal malalignment and, respectively (Tables 4, 5 and 6). No evidence of publication bias was observed in funnel plots.

**Table 3:** Results of quality assessment using Newcastle-Ottawa Scale.

Authors	Representativeness	Sample size	Non-responsiveness	Tools	Confounding factors	Blinding	Statistics	Total
Park et al. 2015	1	1		2	1	2	1	8
Haber et al. 2015	1	1	-	2	1	2	1	8
Yang et ql. 2013	1	0	-	2	1	2	1	7
Mahaudens et al. 2005	1	0	-	2	1	2	1	7
Yazdani et al. 2015	1	0	-	2	1	2	1	7
Lao et al. 2001	1	0	-	2	1	2	1	7
Wu et al. 2019	1	1	-	2	1	2	1	8
Chen et al. 1998	1	1	-	2	1	2	1	8
Mahaudens et al. 2009	1	1	-	2	1	2	1	8
Tekin et al. 2023	1	0	-	2	1	1	1	6
Haddas et al. 2018	1	0	-	2	1	2	1	7
Engsberg eta al. 2003	1	0	-	2	0	2	1	6
Alazzawi et al. 2023	1	0	-	2	1	2	1	7
Bortone et al. 2020	1	0	-	2	1	2	1	7
Delpierre et al. 2019	1	1		2	1	2	1	8
Schmid et al. 2016	1	0	-	2	1	2	1	7
Huysmans et al. 2024	1	1	-	2	1	2	1	8
Mar et al. 2020	1	1		2	1	2	1	8
yagi et al. 2016	1	1	-	2	1	2	1	8
Rebeyrat et al. 2022	1	1	-	2	0	2	1	7
Kawkabani et al. 2021	1	1	-	2	1	2	1	8
Severijn et al. 2021	1	1	-	2	1	2	1	8
Semaan et al. 2022	1	1	-	2	1	2	1	8
Nassim et al. 2024	1	1	-	2	1	2	1	8
Lewis et al. 2015	0	0	-	2	1	2	1	6
Sinaki et al. 2005	1	0		2	1	2	1	7
Saha et al. 2008	0	0	-	2	1	2	1	6
Sarwahi et al. 2002	1	0	-	2	1	2	1	7
Igawa et al. 2022	1	0	-	2	1	2	1	7
Gariepy et al.2009	1	0	-	2	1	2	1	7
Mahaudens et al. 2014	1	0	-	2	0	2	1	6
Nadi et al. 2018	1	0	-	2	1	2	1	7
Garg et al. 2021	1	1	-	2	1	2	1	8
Suh et al. 2023	1	1	-	2	1	2	1	8
Wu et al. 2020	1	0	-	2	1	2	1	7
Total								7.28

Supplementary file 1. GRADE system quality of evidence for scoliosis.

Supplementary file 2. GRADE system quality of evidence for ASD.

Supplementary file 3. GRADE system quality of evidence for Sagittal malalignment.

### Outcomes

### Scoliosis

**Fig. 2:**
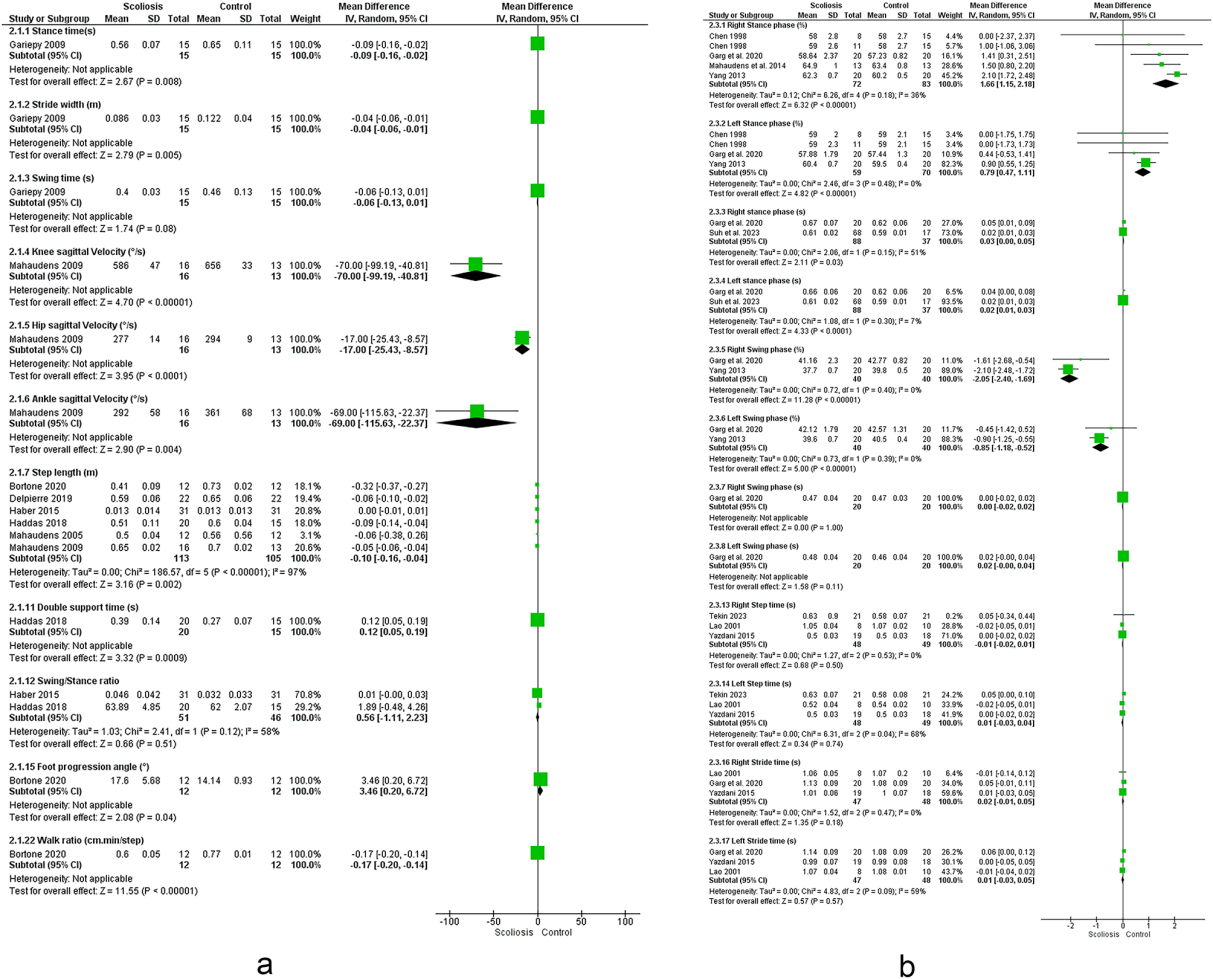

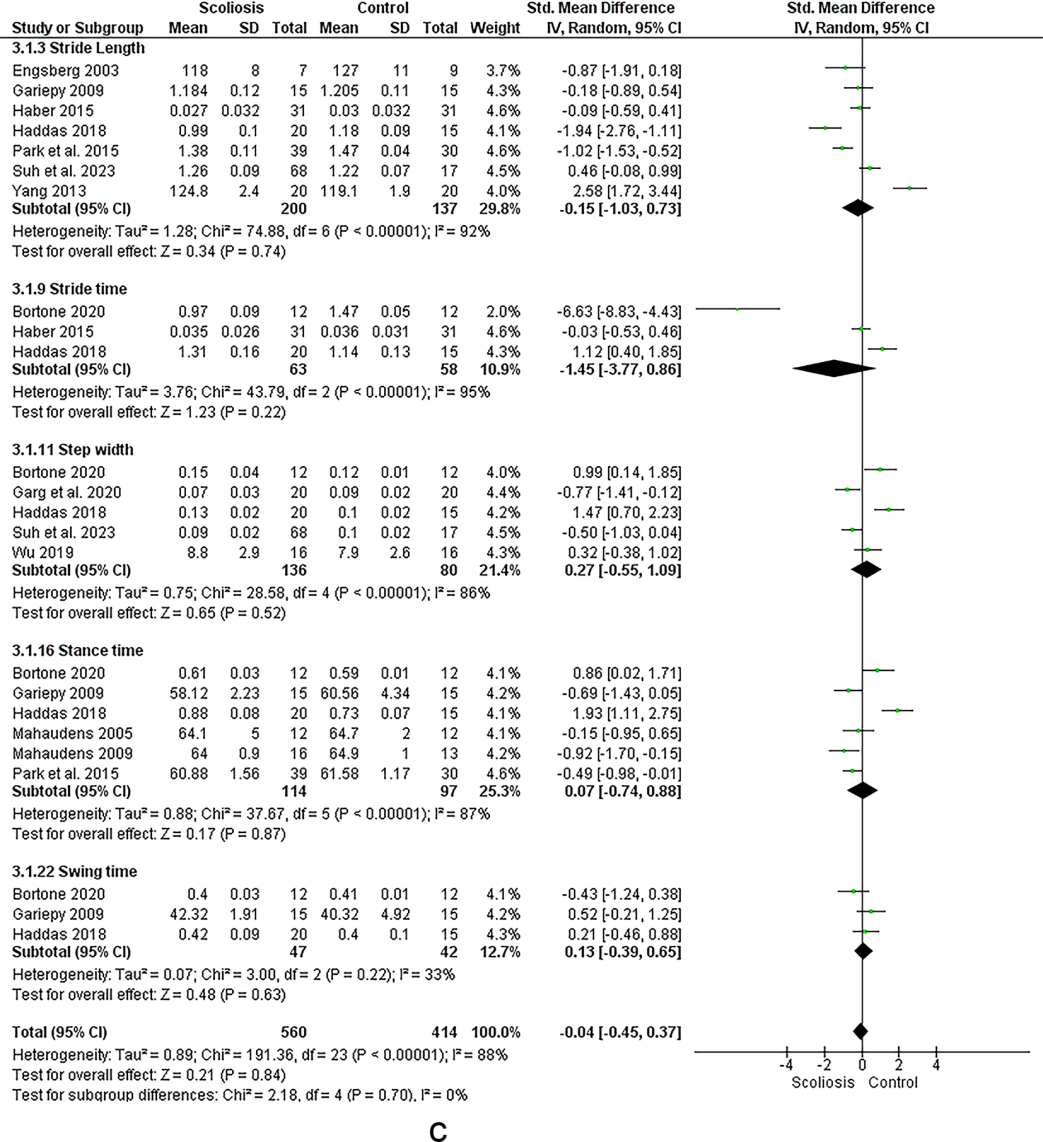

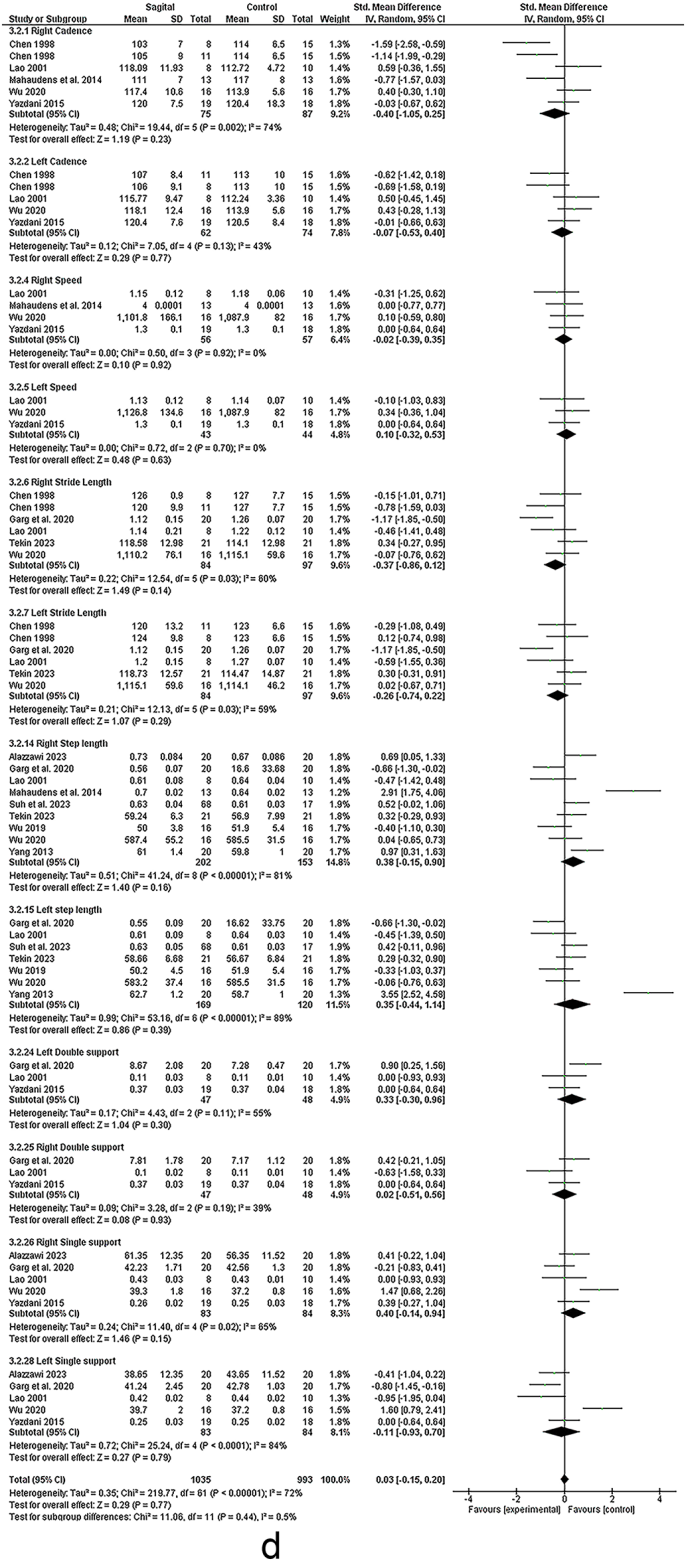

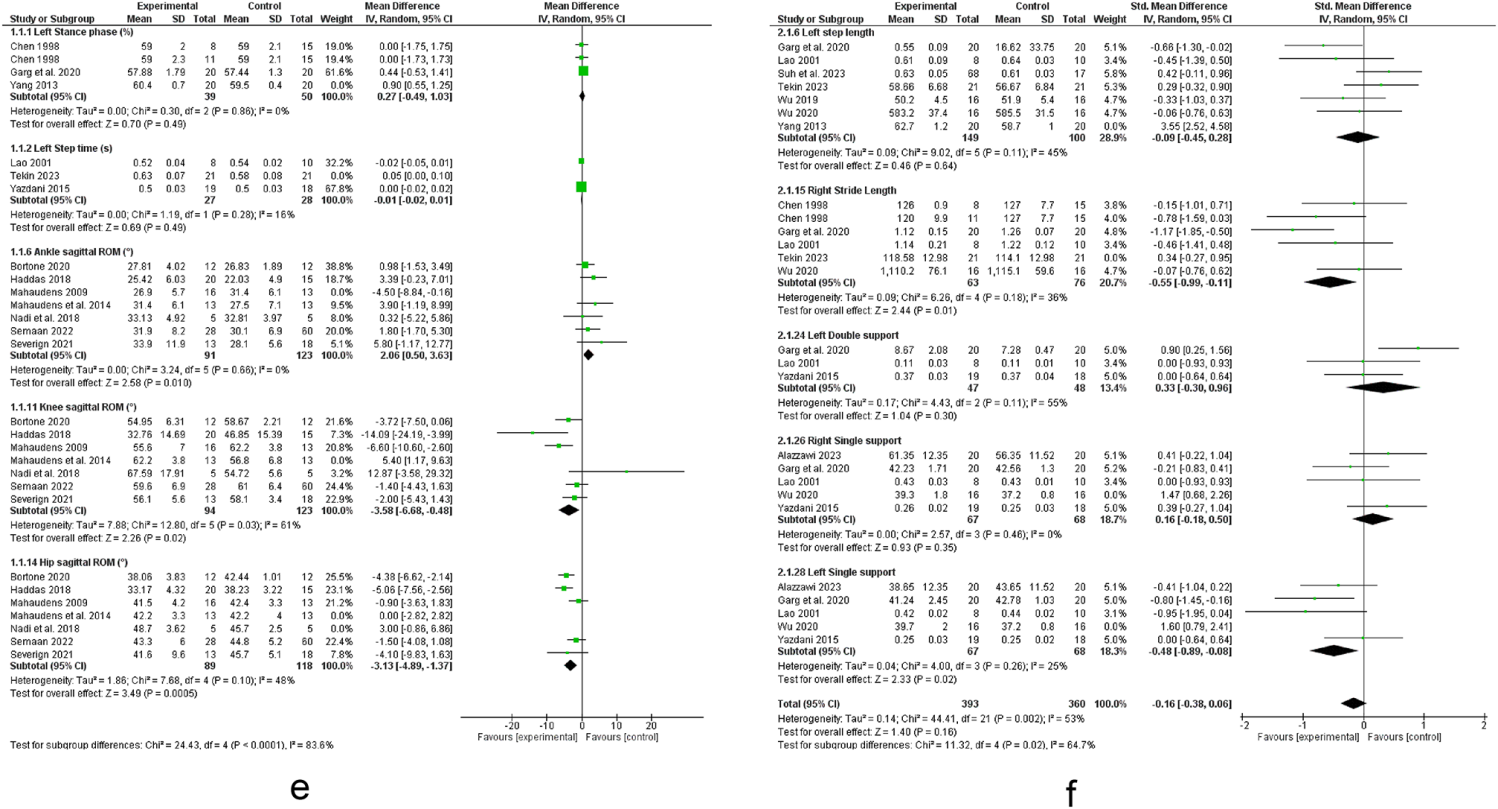
Results of the effect of scoliosis on spatiotemporal parameters (a-d), results for sensitivity analysis with studies removed (e, f).

**Fig. 3:**
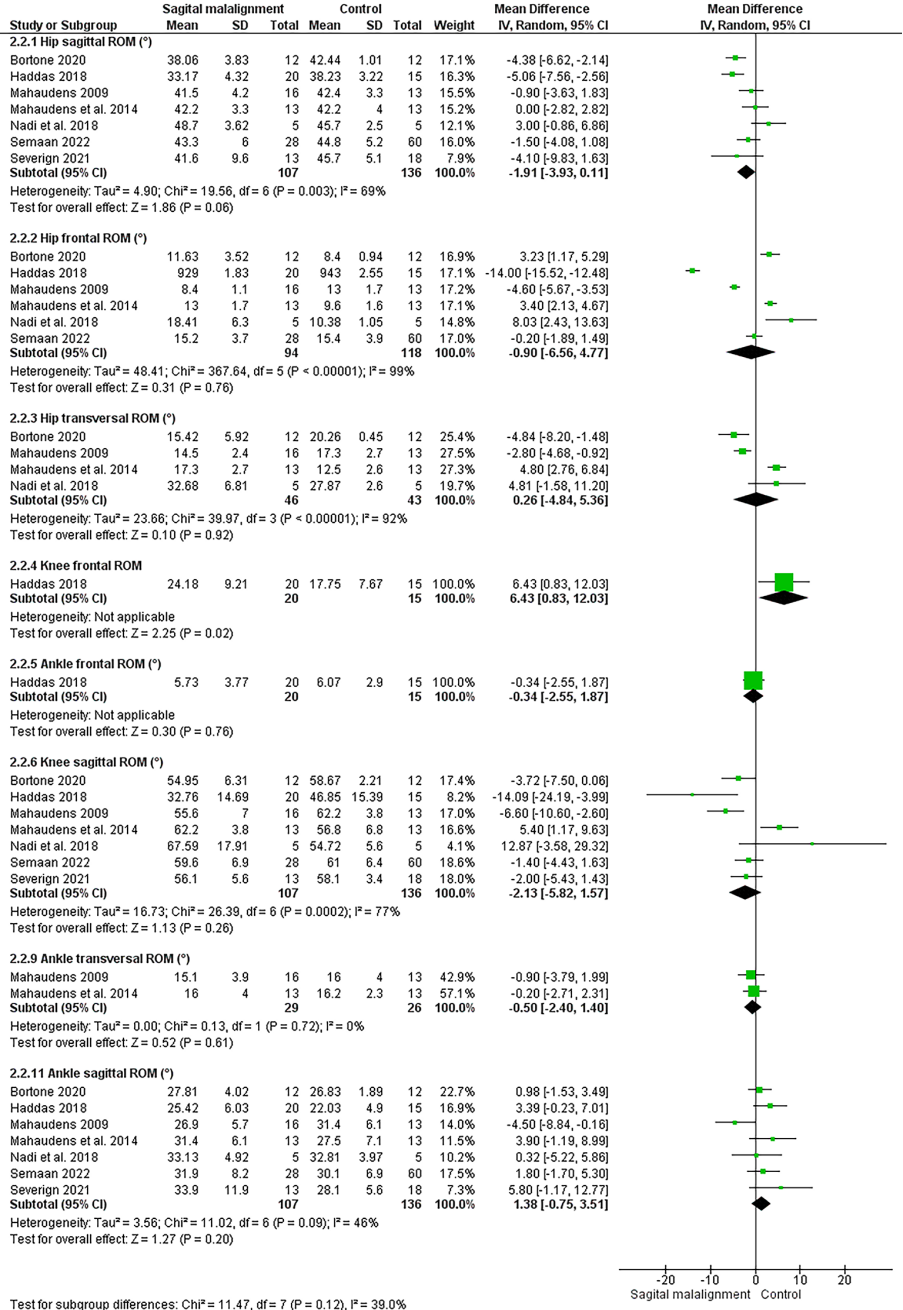
Results of the effect of scoliosis on lower limb joints angles

Twenty five studies investigated the relationship between scoliosis and lower limb kinematics (see Figs. 2 and 3)^12,14,18–20,22,30–48^.

### Spatiotemporal

The meta-analysis suggested strong evidence of a significant increase in right^31,40,42,46^ and left leg stance % ^31,46,49^. However, sensitivity analysis by removing the study by Yang et al. 2013 ^46^ revealed moderate evidence of a non-significant increase in left leg stance time.

The meta-analysis suggested strong evidences of non-significant changes in step and stride time^19,40,48^, left leg cadence^19,31,44,48^, right and left leg speed^19,42,48^, double support % ^19,48,49^, and right leg single support % ^19,44,48–50^. However, sensitivity analysis by removing the study by Tekin et al. 2023 ^32^ revealed moderate non-significant change in left leg step time. Sensitivity analysis by removing the study by Lao 2001 ^48^ revealed moderate evidence of non-significant change in left leg double support. Regarding right single support, removing studies by Wu et al. 2020 ^51^ and Garg et al. 2020 ^49^ resulted in strong evidence.

Moreover, there were moderate evidence of decreased step length^14,20,33,37,45,47^, right leg swing % ^46,49^ and increased stance phase time^39,40^. However, sensitivity analysis by removing the study by Mahaudens et al. 2009 ^14^ revealed a non-significant change in Step length.

Besides, evidence suggested moderate evidence of a non-significant change in swing/stance ratio^33,45^ and swing % ^43^.

Besides, there were conflicting evidence regarding speed^14,20,30,33,34,37,39,40,43,45–47^, cadence^14,20,30,33,34,37,39,40,43,45–47^, stride length^31,32,48^ and time^33,39,43,45,47^, step width^30,33,39,40,47^, stance % ^14,20,33,43,47,52^, right leg cadence^19,31,42,48^ and right leg step^20,33,37,45,47,52^ and stride length^32,39,42,46,48^, left leg step length^30,32,39,40,46,48^ and left leg single support % ^19,44,48–50^. However, sensitivity analysis by removing the study by Tekin et al. 2023 ^32^ revealed strong evidence of a significant decrease in right leg stride length and removing the study by Yang et al. 2013 ^46^ leaded to strong evidence of a non-significant change in left leg step length. Moreover, removing the study by Wu et al. 2020 ^51^ revealed strong evidence of a significant decrease in left leg single support.

### Angles

Meta analysis suggested strong evidence of non-significant increase in ankle sagittal ROM^12,14,22,33,41,42,47^. Sensitivity analysis by the study by Mahaudens et al. (2009) revealed strong evidence of a significant increase in ankle sagittal ROM.

Moderate evidence suggested a non-significant decrease in ankle transverse ROM^14,42^. Furthermore, Conflicting evidence suggested a non-significant change in hip ROM in 3 planes and knee sagittal ROM^12,14,22,33,41,42,47^. However, Sensitivity analysis by removing the study by Mahaudens et al. 2014 ^55^ and Nadi et al. 2018 ^41^ revealed significant decrease in hip sagittal ROM. Removing the study by Mahaudens et al. 2014 ^55^ revealed moderate evidence of significant decrease in knee sagittal ROM. Besides, there was limited evidence of significant increase in knee frontal ROM^33^.

### Adult spinal deformity

#### Spatiotemporal

**Fig. 4:**
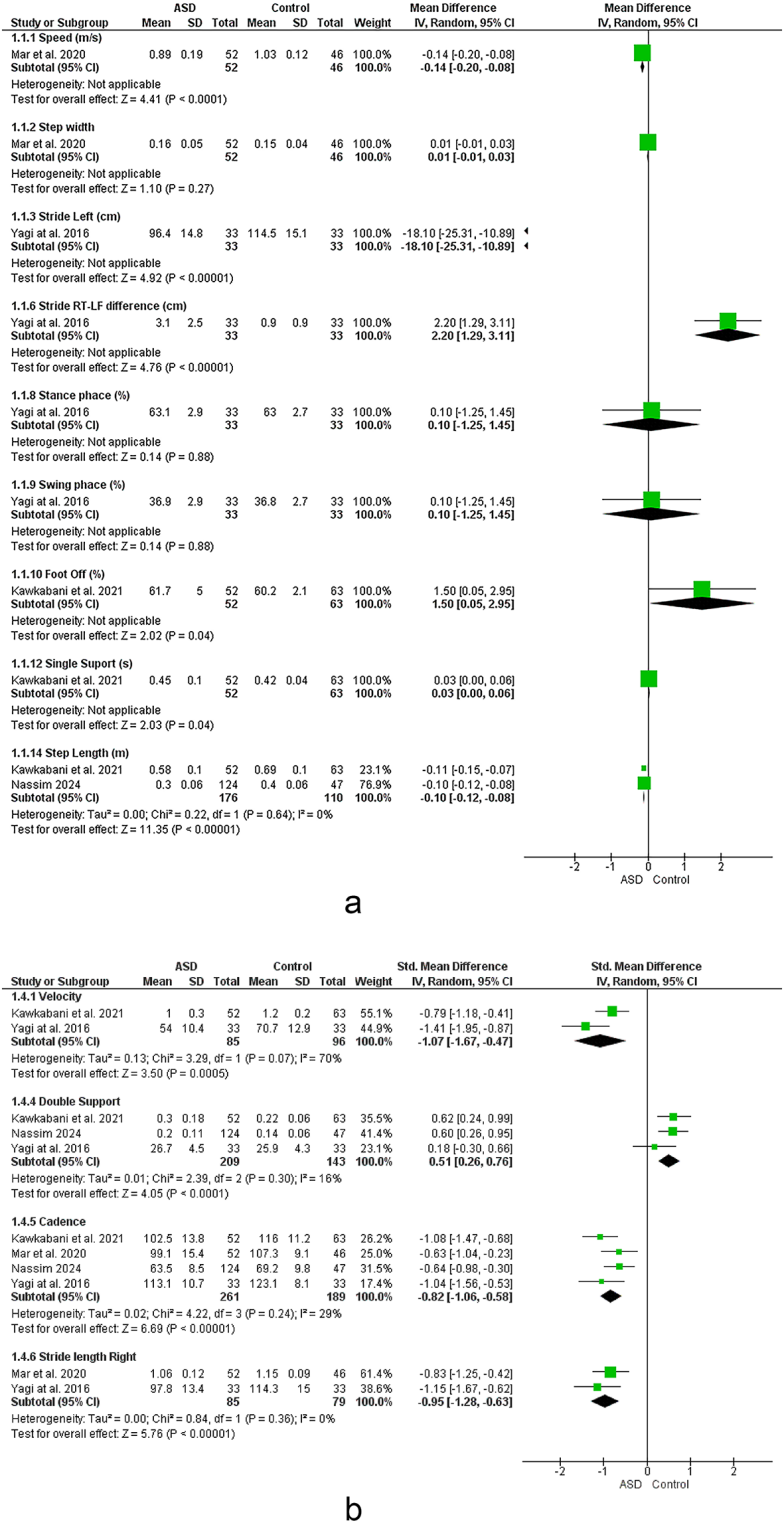
Results of the effect of ASD on spatiotemporal

**Fig. 5:**
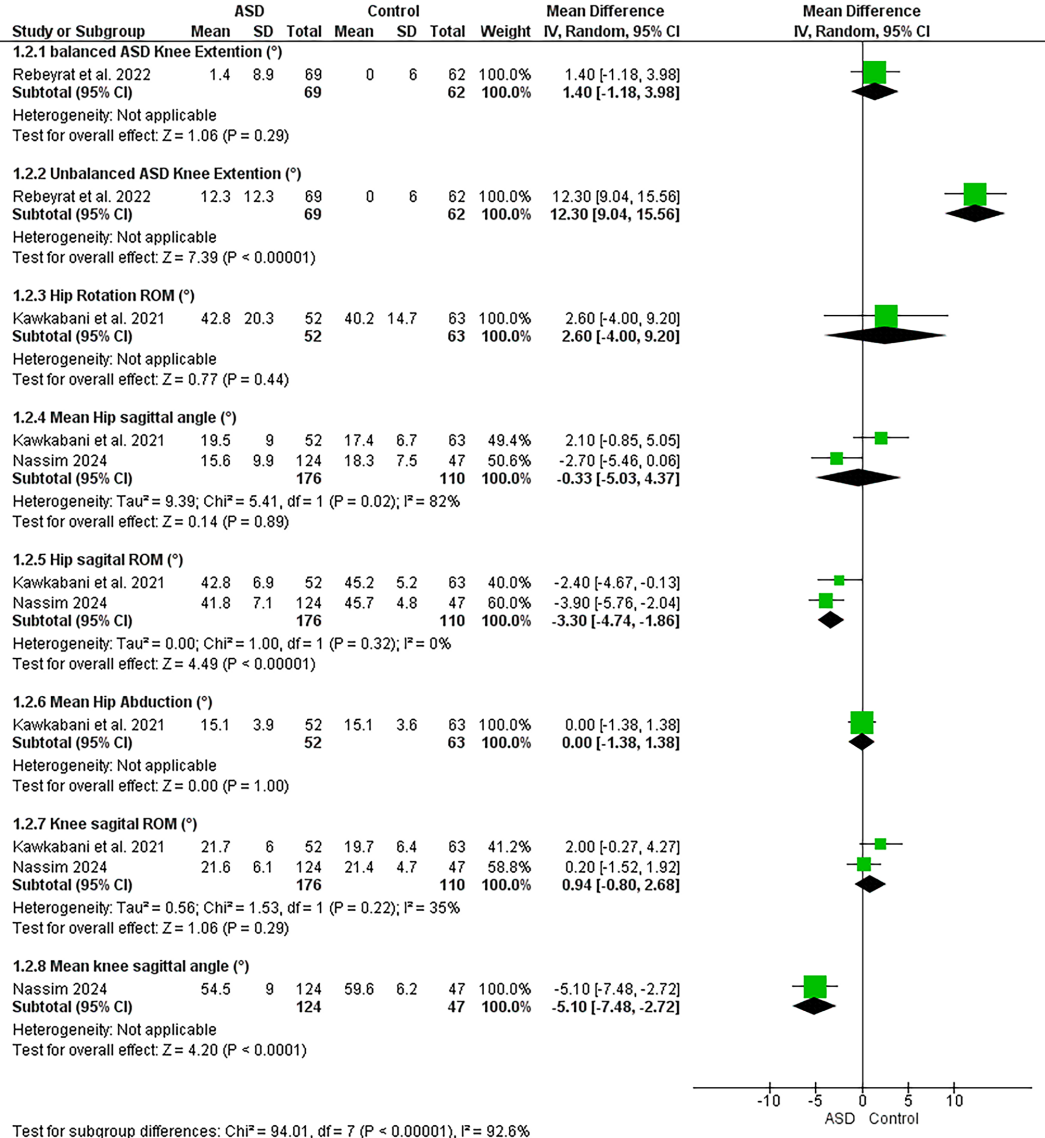
Results of the effect of ASD on lower limb joints angles

Six studies analyzed the effect of ASD and lower limb kinematics (see Figs. 4 and 5)^17,22,53–56^; The meta-analysis suggested strong evidence of a significant increase in double support^53,54,56^ and a significant decrease in cadence^17,53,54,56^ in ASD individual compared to controls. Moreover, moderate evidence suggested a significant decrease in velocity, step length^53,57^ and right leg stride length^17,53^. Limited evidence suggested decreased left leg stride^53^ and speed^17^, and increased foot off % ^62^ and right-left stride difference^53^ in ASD vs. control group.

#### Angles

The meta-analysis suggested significant decrease in hip sagittal ROM (moderate evidence)^54,56^ and non-significant change in knee sagittal ROM^54,56^. Conflicting evidence suggested non-significant change in mean hip sagittal angle. Limited evidence suggested increased knee extension^55^ and decrease in mean knee sagittal angle^56^ in ASD vs. control.

### Sagittal malalignment

**Fig. 6:**
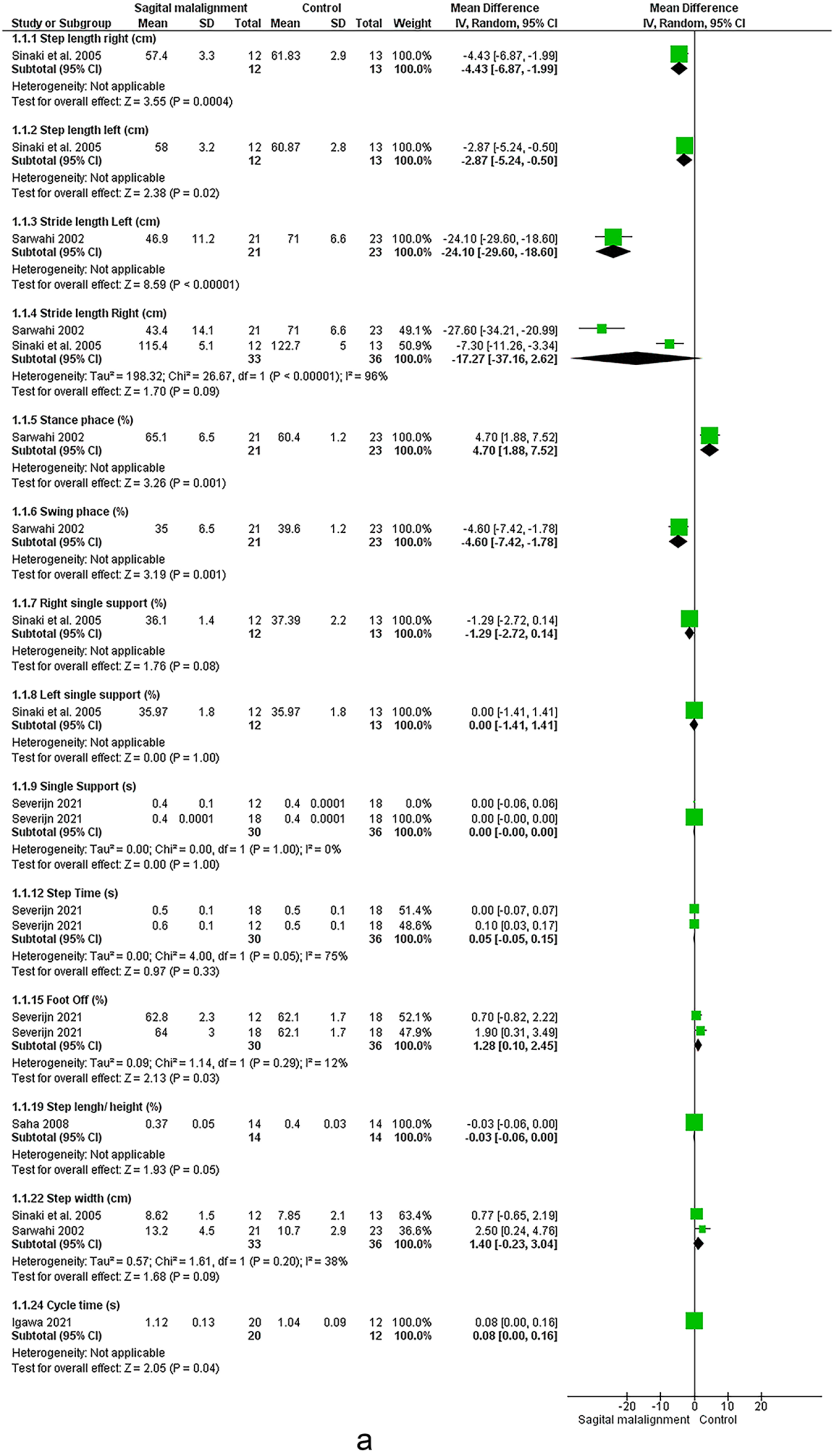

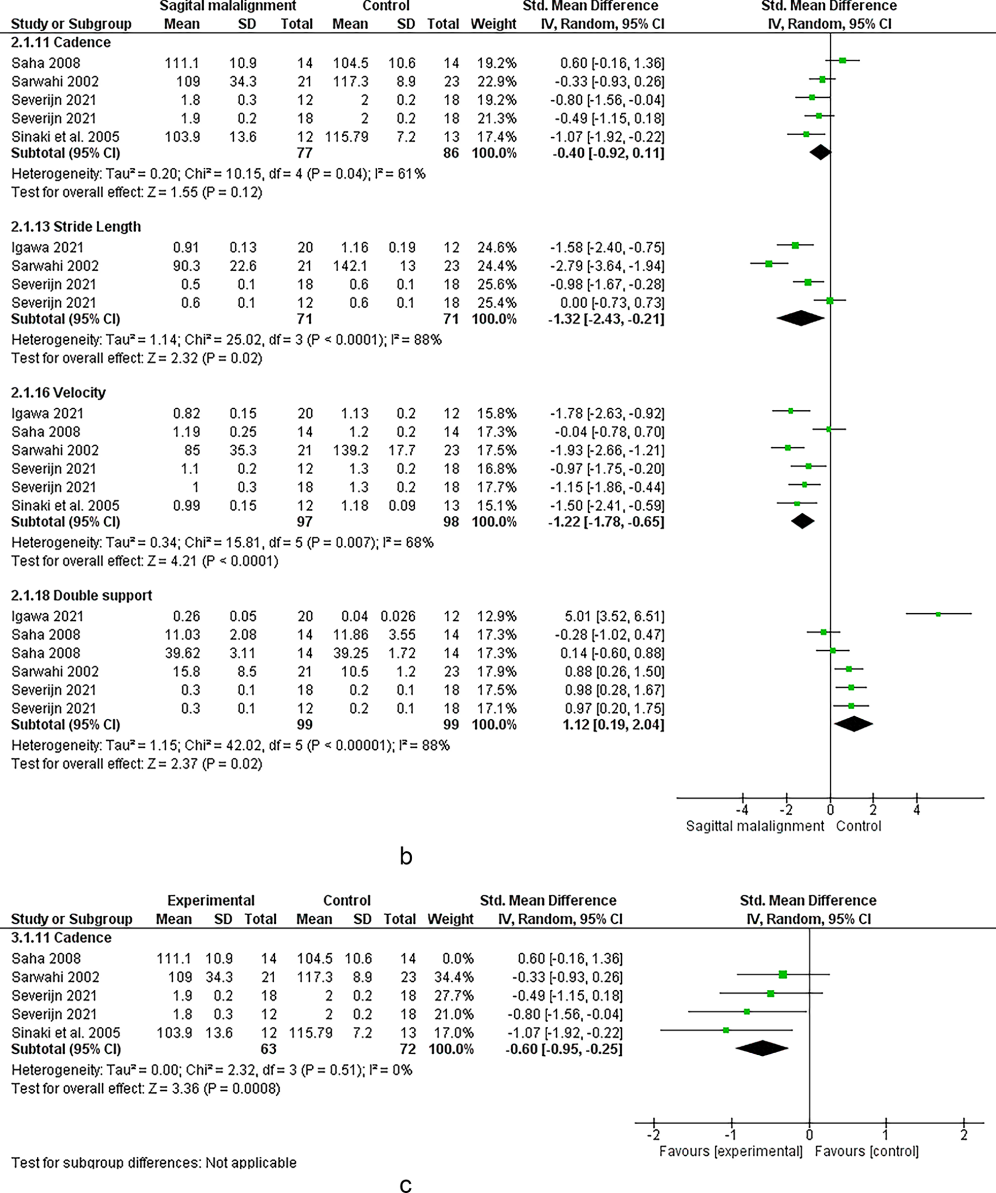
Results of the effect of sagittal malalignment on spatiotemporal (a, b), results of sensitivity analysis (c)

**Fig. 7:**
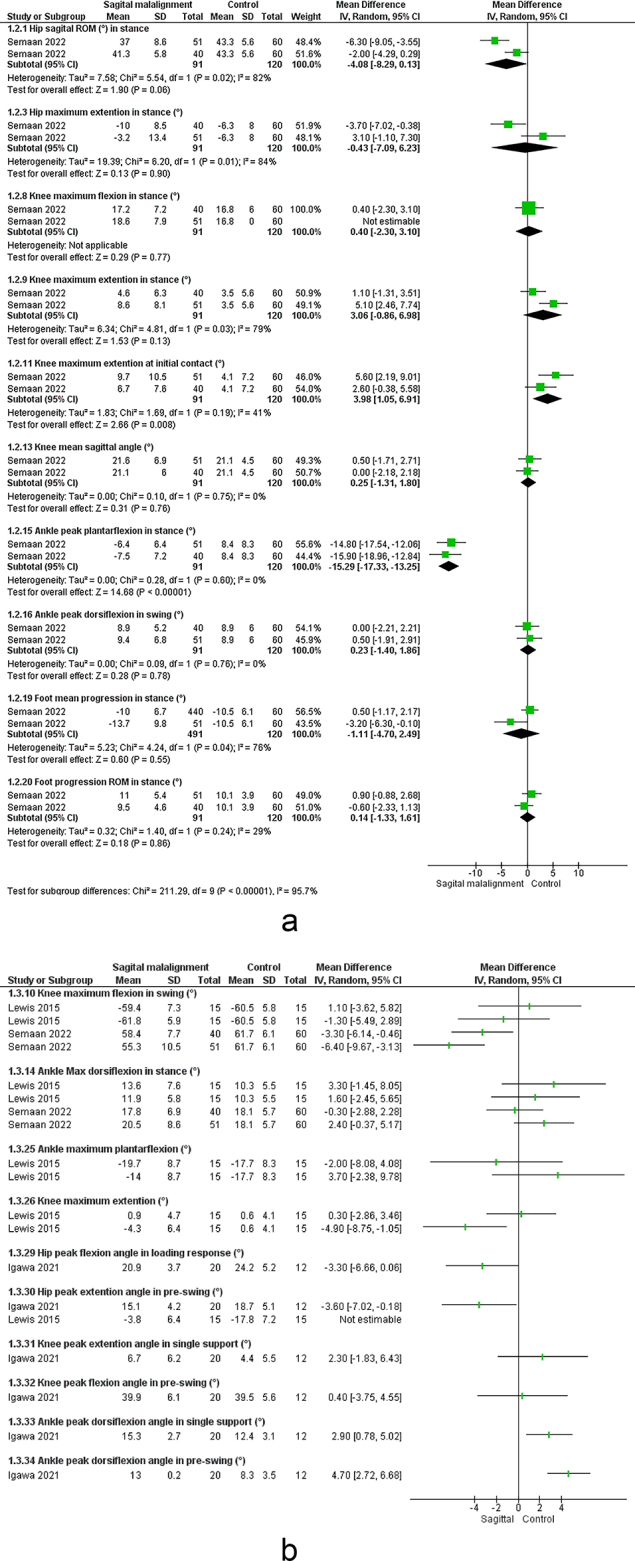

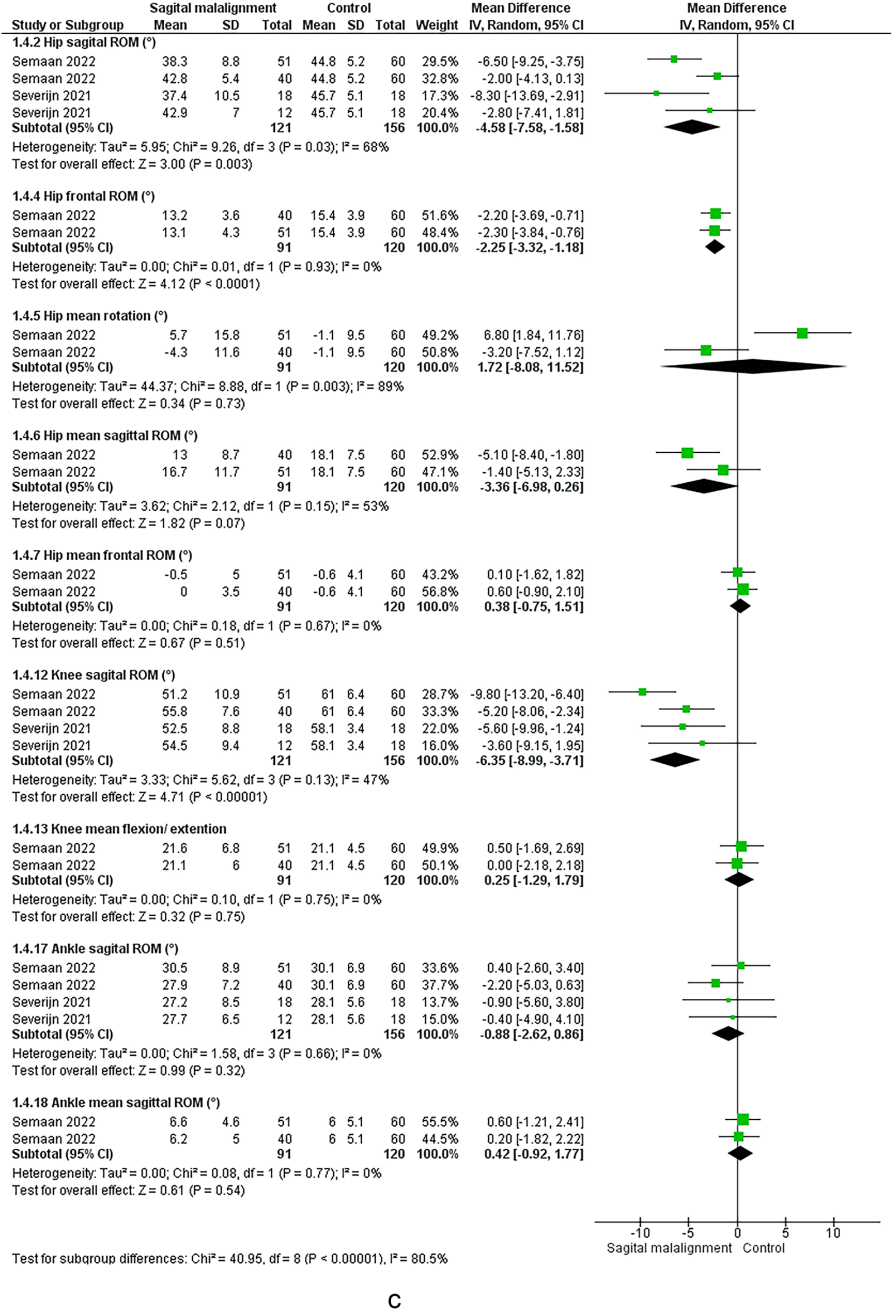
Results of the effect of scoliosis on lower limb joints angles

Seven studies assessed the relationship between spinal sagittal malalignment and lower limb (see Figs. 6 and 7)^12,22,58–62^.

#### Spatiotemporal

Meta-analysis revealed strong evidence significant decrease in velocity^22,59–62^. There were moderate evidence significant increase in double support^22,59–61^, decrease in stride length^22,60,61^ and non-significant change in step width^60,62^. Conflicting evidence suggested decreased right leg stride length^60,62^ and non-significant change in cadence^22,59,60,62^. However, Sensitivity analysis by removing the study by Saha et al. 2008 ^64^ revealed strong evidence of a significant decrease in cadence. Limited evidence suggested increased foot-off % ^22^ in sagittal malalignment vs. control group.

#### Angles

Moderate evidence suggested decreased knee and hip sagittal ROM, and non-significantly changed ankle sagittal ROM^12,22^. Limited evidence suggested significant decrease in hip frontal ROM, ankle plantarflexion ROM in stance phase, and increased knee extension at initial contact. Limited evidence suggested significant decrease in peak knee flexion^12^ and peak hip extension during pre-swing, and increased peak ankle dorsiflexion during pre-swing and single support^61^. There were very limited evidence of peak hip extension in pre-swing and peak knee extension^58^ in sagittal malalignment vs. control group.

## Discussion

We aimed to systematically review the lower limb kinematic differences between those with spinal deformity and healthy controls during gait. We hypothesized that the lower limb kinematic differs between those with spinal deformities and controls during gait.

Three categories of deformities were assessed in the studies, including scoliosis, sagittal malalignment and ASD. Individuals with scoliosis were defined as having a cobb angle of greater than 10°. Those with ASD typically exhibited sagittal plane pelvic tilt exceeding 20°, sagittal vertical axis greater than 40 mm, and a cobb angle greater than 20°. Individuals with sagittal malalignment demonstrated various forms of malalignment in the sagittal plane including dropped head, hyper kyphosis, swayback and flat back.

Sthrong evidence suggested significant increased double support^53,54,56^ and decreased cadence^17,53,54,56^ in ASD vs. control. Strong significant decrease in left leg single support, hip and knee sagittal ROM^12,14,22,33,47^, and non-significant change in right leg single support time, right and left leg step time^19,48^ in scoliosis vs. control. Strong evidence suggested significant decrease in velocity^22,59–62^ in sagittal malalignment group vs. control.

### Scoliosis

The meta-analysis reveals significant deviations in key spatiotemporal parameters among individuals with scoliosis. Increased right and left leg stance time percentages were strongly evident, suggesting compensatory strategies to enhance stability during ambulation. However, sensitivity analyses indicate that the significance of these findings varies depending on the removal of Yang et al. (2013) which rendered results for left leg stance non-significant. The potential reason might be the difference between participants scoliosis type and degree and position of the curve in this study. During movement, these spinal deformities can interfere with lower limb biomechanics via a kinematic chain and fascial slings^14^, resulting in compensatory adjustments on the opposite side^15^. These finding highlight the asymmetry in gait mechanics, likely influenced by the curvature of the spine, and underscore the need for targeted interventions to address side-specific deficits, particularly on the left leg side, to improve overall gait stability and reduce fall risk. However, the included studies did not assess gait mechanics of the lower limbs on the convex and concave sides of the scoliosis curvature, which is a notable limitation. Future research should address this gap to provide a more comprehensive understanding of scoliotic gait dynamics.

Interestingly, there was no significant change observed in step and stride time, cadence, speed, double support, or single support for most cases, indicating that while scoliosis alters stability-related parameters, overall gait rhythm and timing might remain relatively preserved. This suggests a degree of adaptability in neural control mechanisms, allowing individuals to maintain a consistent walking rhythm despite structural challenges. However, changes in step time, single and double support observed in the sensitivity analysis may be attributed to variations in curve type in different studies, as scoliosis is diverse and can differ significantly between cases. More targeted research is needed that categorizes participants into distinct groups based on the type, degree, and location of spinal curvature. Additionally, it is crucial to report results separately for the convex and concave sides to gain a comprehensive understanding of how scoliosis affects gait dynamics.

Moderate evidence suggested a decreased step length in individuals with scoliosis which may negatively impact locomotor efficiency by reducing stride length and speed. This shorter step length reflects a cautious gait strategy aimed at reducing instability. Coupled with increased stance phase time, these findings imply a compensatory mechanism to ensure stability at the expense of efficiency. However, sensitivity analysis, such as the exclusion of Mahaudens et al. (2009), revealed non-significant results, further emphasizing variability across studies and populations.

The analysis also indicated moderate non-significant changes in the swing/stance ratio and swing percentage, further demonstrating the compensatory adjustments in gait mechanics. These parameters suggest that while scoliosis affects certain gait characteristics, others, such as rhythm and timing, remain unaffected, potentially preserving functional mobility.

The results reveal conflicting evidence regarding speed, cadence, stride length, and time, as well as other parameters like step width and stance percentage. This variability may stem from differences in scoliosis severity, participant age, and methodological heterogeneity across studies. These findings emphasize the importance of standardizing methodologies in future research to achieve more conclusive results.

Strong evidence pointed to non-significant changes in ankle sagittal ROM, sensitivity analysis showed strong evidence of decrease in this parameter indicating variability dependency on differences in ages or position and degree of curve in these studies.

Limited evidence suggested a significant increase in knee frontal ROM, which could indicate compensatory movements to maintain stability in response to scoliosis-related asymmetry. Conflicting evidence was observed for changes in hip ROM in all planes and knee sagittal ROM, with sensitivity analyses revealing significant findings when certain studies were excluded. For example, removing Mahaudens et al. (2014) or Nadi et al. (2018) led to significant results for hip and knee sagittal ROM, indicating that scoliosis may influence dynamic control of these joints in a subset of individuals. This variability in sensitivity analysis is probably due to the age variabilities included in meta-analysis for these outcomes.

From a clinical perspective, these insights underscore the importance of individualized interventions aimed at addressing side-specific deficits, improving step length, and enhancing joint mobility. Rehabilitation programs focusing on strengthening postural control and improving stability, particularly on the left side, may help optimize gait performance and reduce fall risk. Further research should aim to resolve the conflicting evidence by exploring potential mediating variables such as age or curve type that could influence outcomes. Additionally, it is crucial to investigate how these gait alterations impact long-term mobility and fatigue.

### Adult spinal deformity

The findings from our meta-analysis indicate distinct patterns in lower limb kinematics among individuals with ASD when compared to control groups. The strong evidence supporting increased double support time and decreased cadence suggests a unique and more cautious gait characteristic. This indicates compensatory mechanisms to improve balance and stabilizing COM^12^ and reduce risk of falls^54^.

Similarly, the significant decrease in cadence, alongside moderate evidence of reduced velocity and step length, underscores a potential trade-off in speed for stability which may be due to joint stiffness or weakness^63^. These findings illuminates how these individuals may be adapting their locomotion strategies in the face of inherent motor planning and execution challenges.

The findings related to hip and knee sagittal ROM contribute to our understanding of joint mechanics in this population. The significant reduction in hip sagittal ROM reinforces the idea that individuals with ASD may exhibit a more restricted range of movement during ambulation. Conversely, the lack of a significant change in knee sagittal ROM juxtaposed with the conflicting evidence regarding the knee’s mean sagittal angle points to the complexity of lower limb biomechanics in ASD. It raises important questions about whether adaptations in gait mechanics are uniform across all joints or if there exists a compensatory hierarchy where certain joint movements are preserved while others are altered.

## Sagittal malalignment

Our meta-analysis presents strong evidence for a significant decrease in gait velocity, which is consistent with prior literature suggesting that sagittal malalignments impair propulsion and overall walking efficiency^22^. This reduction in velocity may indicate a compensatory adaptation where individuals with malalignments adopt a more cautious gait pattern, potentially to maintain stability^12^ and reduce the risk of falls^54^.

In addition to decreased velocity, the moderate evidence for increased double support time highlights a possible shift towards a more stable, yet less dynamic, gait. Prolonged double support may indicate an attempt to enhance balance^12^. Conversely, the observed decrease in stride length further reinforces the notion that individuals with sagittal malalignments may be compensating for instability by shortening their strides, leading to a less effective gait pattern.

Interestingly, the non-significant changes in cadence and step width suggest that while overall gait quality is affected, the rhythm of walking remains relatively stable under these conditions. However, sensitivity analysis showed strong evidence of decreased cadence after removal of the study by Saha et al. which is probably due to its very small sample size. It suggests that scoliosis may indeed impact walking rhythm more than previously understood, highlighting the need for further research to explore the factors contributing to these discrepancies and to standardize gait assessments in this population. The conflicting evidence regarding decreased right leg stride length also suggests that lateral asymmetries may be present in some individuals, highlighting potential areas for focused intervention.

The moderate evidence indicating decreased knee and hip sagittal ROM suggests that individuals with these malalignments may experience constrained movement patterns that affect their overall mobility. The non-significant change in ankle sagittal ROM, however, introduces an element of complexity, indicating that ankle mechanics may remain more intact compared to the hip and knee.

Additionally, the findings of increased peak ankle dorsiflexion during pre-swing and single support phase suggest a compensatory strategy that might be aimed at enhancing foot clearance and avoiding stumbling, possibly due to the altered kinematics imposed by malalignment. The very limited evidence regarding peak hip extension and peak knee extension during pre-swing indicates a need for further investigation into these critical phases of gait, as they are pivotal for effective locomotion and energy transfer.

### Limitations and recommendations for Future studies

This review on gait patterns in individuals with scoliosis identifies key limitations. Population variability necessitates the consideration of age, sex, and height in analyses, as differences in these factors may significantly influence outcomes. Therefore, future research should account for age, sex, and height when analyzing data, considering them either as controlled variables or as effect modifiers in statistical analyses such as two-way ANOVA. A key limitation of our study is the complexity of sagittal malalignment, which includes various spinal and postural disorders that were not incorporated into the meta-analysis. This prevents us from drawing strong conclusions regarding its effects on lower limb kinematics. Additionally, more research on ASD is necessary to strengthen our findings and improve clinical insights. Small sample sizes in certain studies^18,34,41,42,47,48,59,62,64^ may compromise the validity of their results. To ensure a sample size that provides acceptable statistical power, future research should incorporate sample size calculations utilizing G*Power or standard formulas. Future studies should improve gait analysis methodologies, especially regarding marker visibility and asymmetrical gait. Standardizing measurement techniques will improve reliability due to potential projection errors in measurements. Scoliosis includes a variety of curve types, and focusing only on specific ones may not accurately reflect the entire scoliosis population. It’s important to consider a broader range of curve types to better understand this condition. Moreover, the convex-concave sides were not considered in the studies included. Future research should address this issue in the inclusion criteria. Addressing these issues requires standardizing techniques, dissociating various curve types, reporting the convex side, matching groups, increasing the sample sizes to facilitate early identification of gait abnormalities and inform individualized rehabilitation strategies.

## Conclusion

This systematic review and meta-analysis revealed significant differences in lower limb kinematics during gait between individuals with spinal deformities and healthy controls. These gait alterations are likely compensatory mechanisms aimed at enhancing balance and stability. The findings underscore the importance of individualized rehabilitation strategies to improve gait efficiency and mobility, particularly considering the complexity of spinal deformities. Future research should address existing limitations, such as small sample sizes and group heterogeneity, by standardizing measurement techniques and incorporating a broader range of spinal conditions. By integrating clinical outcomes with laboratory data, clinicians can deepen their understanding of gait alterations and develop more effective interventions for spinal deformities.

### Data Availability

All data generated or analyzed during this study were included in this published article (and its Supplementary Information files).

### CRediT authorship contribution statement

**Fateme Khorramroo**: Conceptualizing, Methodology, Software, Validation, Investigation, Data Curation, Writing Original Draft, Review & Editing, Supervision, Project Administration. **Seyed Hamed Mousavi**: Conceptualizing, Methodology, Validation, Formal analysis, Investigation, Data Curation, Review & Editing, Supervision, Project Administration. **Reza Rajabi**: Conceptualizing, Validation, Methodology, Review & Editing, Data Curation, Formal analysis, Investigation.

## Electronic supplementary material

Below is the link to the electronic supplementary material.

Supplementary Material 1

Supplementary Material 1

Supplementary Material 1

## Data Availability

All data generated or analysed during this study are included in this published article (and its Supplementary Information files).
