# Supplementary material for: Effects of spinal deformities on lower limb kinematics during walking: a systematic review and meta-analysis
Source: Sci Rep. 2025 Feb 7;15:4608. doi: 10.1038/s41598-025-88886-5 (PMC11806027; doi:10.1038/s41598-025-88886-5)
Supplement: Supplementary file 1 — Supplementary Material 1 [file 41598_2025_88886_MOESM1_ESM.docx]

Scoliosis compared to control

| **Certainty assessment** | | | | | | | **№ of patients** | | **Effect** | | **Certainty** | **Importance** |
| --- | --- | --- | --- | --- | --- | --- | --- | --- | --- | --- | --- | --- |
| **№ of studies** | **Study design** | **Risk of bias** | **Inconsistency** | **Indirectness** | **Imprecision** | **Other considerations** | **Scoliosis** | **Control** | **Relative (95% CI)** | **Absolute (95% CI)** |  |  |
| **Spatiotemporal - Stance time(s)** | | | | | | | | | | | | |
| 1 | non-randomised studies | not serious | not serious | not serious | serious | strong association all plausible residual confounding would reduce the demonstrated effect dose response gradient | 15 cases 15 controls | | **RR -0.09** (-0.16 to -0.02) | - | ⨁⨁⨁⨁ High |  |
|  |  |  |  |  |  |  | - | 0.0% |  | **0 fewer per 1,000** (from 0 fewer to 0 fewer) |  |  |
| **Spatiotemporal - Stride width (m)** | | | | | | | | | | | | |
| 1 | non-randomised studies | not serious | not serious | not serious | serious | strong association all plausible residual confounding would reduce the demonstrated effect dose response gradient | 15 cases 15 controls | | **RR -0.04** (-0.06 to -0.01) | - | ⨁⨁⨁⨁ High |  |
|  |  |  |  |  |  |  | - | 0.0% |  | **0 fewer per 1,000** (from 0 fewer to 0 fewer) |  |  |
| **Spatiotemporal - Swing time (s)** | | | | | | | | | | | | |
| 1 | non-randomised studies | not serious | not serious | not serious | serious | all plausible residual confounding would reduce the demonstrated effect dose response gradient | 15 cases 15 controls | | **RR -0.06** (-0.13 to 0.01) | - | ⨁⨁⨁◯ Moderate |  |
|  |  |  |  |  |  |  | - | 0.0% |  | **0 fewer per 1,000** (from 0 fewer to 0 fewer) |  |  |
| **Spatiotemporal - Knee sagittal Velocity (°/s)** | | | | | | | | | | | | |
| 1 | non-randomised studies | not serious | not serious | not serious | serious | very strong association all plausible residual confounding would reduce the demonstrated effect dose response gradient | 16 cases 13 controls | | **RR -70.00** (-99.19 to -40.81) | - | ⨁⨁⨁⨁ High |  |
|  |  |  |  |  |  |  | - | 0.0% |  | **0 fewer per 1,000** (from 0 fewer to 0 fewer) |  |  |
| **Spatiotemporal - Hip sagittal Velocity (°/s)** | | | | | | | | | | | | |
| 1 | non-randomised studies | not serious | not serious | not serious | serious | very strong association all plausible residual confounding would reduce the demonstrated effect dose response gradient | 16 cases 13 controls | | **RR -17.00** (-25.43 to -8.57) | - | ⨁⨁⨁⨁ High |  |
|  |  |  |  |  |  |  | - | 0.0% |  | **0 fewer per 1,000** (from 0 fewer to 0 fewer) |  |  |
| **Spatiotemporal - Ankle sagittal Velocity (°/s)** | | | | | | | | | | | | |
| 1 | non-randomised studies | not serious | not serious | not serious | serious | very strong association all plausible residual confounding would reduce the demonstrated effect dose response gradient | 16 cases 13 controls | | **RR -69.00** (-115.63 to -22.37) | - | ⨁⨁⨁⨁ High |  |
|  |  |  |  |  |  |  | - | 0.0% |  | **0 fewer per 1,000** (from 0 fewer to 0 fewer) |  |  |
| **Spatiotemporal - Step length (m)** | | | | | | | | | | | | |
| 6 | non-randomised studies | not serious | very serious | not serious | serious | strong association all plausible residual confounding would reduce the demonstrated effect dose response gradient | 113 cases 105 controls | | **RR -0.10** (-0.16 to -0.04) | - | ⨁⨁◯◯ Low |  |
|  |  |  |  |  |  |  | - | 0.0% |  | **0 fewer per 1,000** (from 0 fewer to 0 fewer) |  |  |
| **Spatiotemporal - Double support time (s)** | | | | | | | | | | | | |
| 1 | non-randomised studies | not serious | not serious | not serious | serious | very strong association all plausible residual confounding would reduce the demonstrated effect dose response gradient | 20 cases 15 controls | | **RR 0.12** (0.05 to 0.19) | - | ⨁⨁⨁⨁ High |  |
|  |  |  |  |  |  |  | - | 0.0% |  | **0 fewer per 1,000** (from 0 fewer to 0 fewer) |  |  |
| **Spatiotemporal - Swing/Stance ratio** | | | | | | | | | | | | |
| 2 | non-randomised studies | not serious | not serious | not serious | serious | all plausible residual confounding would reduce the demonstrated effect dose response gradient | 51 cases 46 controls | | **RR 0.56** (-1.11 to 2.23) | - | ⨁⨁⨁◯ Moderate |  |
|  |  |  |  |  |  |  | - | 0.0% |  | **0 fewer per 1,000** (from 0 fewer to 0 fewer) |  |  |
| **Spatiotemporal - Foot progression angle (°)** | | | | | | | | | | | | |
| 1 | non-randomised studies | not serious | not serious | not serious | serious | strong association all plausible residual confounding would reduce the demonstrated effect dose response gradient | 12 cases 12 controls | | **RR 3.46** (0.20 to 6.72) | - | ⨁⨁⨁⨁ High |  |
|  |  |  |  |  |  |  | - | 0.0% |  | **0 fewer per 1,000** (from 0 fewer to 0 fewer) |  |  |
| **Spatiotemporal - Walk ratio (cm.min/step)** | | | | | | | | | | | | |
| 1 | non-randomised studies | not serious | not serious | not serious | serious | very strong association all plausible residual confounding would reduce the demonstrated effect dose response gradient | 12 cases 12 controls | | **RR -0.17** (-0.20 to -0.14) | - | ⨁⨁⨁⨁ High |  |
|  |  |  |  |  |  |  | - | 0.0% |  | **0 fewer per 1,000** (from 0 fewer to 0 fewer) |  |  |
| **Angle - Hip sagittal ROM (°)** | | | | | | | | | | | | |
| 7 | non-randomised studies | not serious | serious | not serious | serious | all plausible residual confounding would reduce the demonstrated effect dose response gradient | 107 cases 136 controls | | **RR -1.91** (-3.93 to 0.11) | - | ⨁⨁◯◯ Low |  |
|  |  |  |  |  |  |  | - | 0.0% |  | **0 fewer per 1,000** (from 0 fewer to 0 fewer) |  |  |
| **Angle - Hip frontal ROM (°)** | | | | | | | | | | | | |
| 6 | non-randomised studies | not serious | very serious | not serious | serious | all plausible residual confounding would reduce the demonstrated effect dose response gradient | 94 cases 118 controls | | **RR -0.90** (-6.56 to 4.77) | - | ⨁◯◯◯ Very low |  |
|  |  |  |  |  |  |  | - | 0.0% |  | **0 fewer per 1,000** (from 0 fewer to 0 fewer) |  |  |
| **Angle - Hip transversal ROM (°)** | | | | | | | | | | | | |
| 4 | non-randomised studies | not serious | very serious | not serious | serious | all plausible residual confounding would reduce the demonstrated effect dose response gradient | 46 cases 43 controls | | **RR 0.26** (-4.84 to 5.36) | - | ⨁◯◯◯ Very low |  |
|  |  |  |  |  |  |  | - | 0.0% |  | **0 fewer per 1,000** (from 0 fewer to 0 fewer) |  |  |
| **Angle - Knee frontal ROM** | | | | | | | | | | | | |
| 1 | non-randomised studies | not serious | not serious | not serious | serious | strong association all plausible residual confounding would reduce the demonstrated effect dose response gradient | 20 cases 15 controls | | **RR 6.43** (0.83 to 12.03) | - | ⨁⨁⨁⨁ High |  |
|  |  |  |  |  |  |  | - | 0.0% |  | **0 fewer per 1,000** (from 0 fewer to 0 fewer) |  |  |
| **Angle - Ankle frontal ROM (°)** | | | | | | | | | | | | |
| 1 | non-randomised studies | not serious | not serious | not serious | serious | all plausible residual confounding would reduce the demonstrated effect dose response gradient | 20 cases 15 controls | | **RR -0.34** (-2.55 to 1.87) | - | ⨁⨁⨁◯ Moderate |  |
|  |  |  |  |  |  |  | - | 0.0% |  | **0 fewer per 1,000** (from 0 fewer to 0 fewer) |  |  |
| **Angle - Knee sagittal ROM (°)** | | | | | | | | | | | | |
| 7 | non-randomised studies | not serious | very serious | not serious | serious | all plausible residual confounding would reduce the demonstrated effect dose response gradient | 107 cases 106 controls | | **RR -2.13** (-5.82 to 1.57) | - | ⨁◯◯◯ Very low |  |
|  |  |  |  |  |  |  | - | 0.0% |  | **0 fewer per 1,000** (from 0 fewer to 0 fewer) |  |  |
| **Angle - Ankle transversal ROM (°)** | | | | | | | | | | | | |
| 2 | non-randomised studies | not serious | not serious | not serious | serious | all plausible residual confounding would reduce the demonstrated effect dose response gradient | 29 cases 26 controls | | **RR -0.5** (-2.4 to 1.4) | - | ⨁⨁⨁◯ Moderate |  |
|  |  |  |  |  |  |  | - | 0.0% |  | **0 fewer per 1,000** (from 0 fewer to 0 fewer) |  |  |
| **Angle - Ankle sagittal ROM (°)** | | | | | | | | | | | | |
| 7 | non-randomised studies | not serious | not serious | not serious | serious | all plausible residual confounding would reduce the demonstrated effect dose response gradient | 107 cases 136 controls | | **RR 1.38** (-0.75 to 3.51) | - | ⨁⨁⨁◯ Moderate |  |
|  |  |  |  |  |  |  | - | 0.0% |  | **0 fewer per 1,000** (from 0 fewer to 0 fewer) |  |  |
| **Spatiotemporal RL - Right Stance phase (%)** | | | | | | | | | | | | |
| 4 | non-randomised studies | not serious | not serious | not serious | serious | very strong association all plausible residual confounding would reduce the demonstrated effect dose response gradient | 72 cases 83 controls | | **RR 1.66** (1.15 to 2.18) | - | ⨁⨁⨁⨁ High |  |
|  |  |  |  |  |  |  | - | 0.0% |  | **0 fewer per 1,000** (from 0 fewer to 0 fewer) |  |  |
| **Spatiotemporal RL - Left Stance phase (%)** | | | | | | | | | | | | |
| 3 | non-randomised studies | not serious | not serious | not serious | serious | very strong association all plausible residual confounding would reduce the demonstrated effect dose response gradient | 59 cases 70 controls | | **RR 0.79** (0.47 to 1.11) | - | ⨁⨁⨁⨁ High |  |
|  |  |  |  |  |  |  | - | 0.0% |  | **0 fewer per 1,000** (from 0 fewer to 0 fewer) |  |  |
| **Spatiotemporal RL - Right stance phase (s)** | | | | | | | | | | | | |
| 2 | non-randomised studies | not serious | not serious | not serious | not serious | strong association all plausible residual confounding would reduce the demonstrated effect dose response gradient | 88 cases 37 controls | | **RR 0.03** (0.00 to 0.05) | - | ⨁⨁⨁⨁ High |  |
|  |  |  |  |  |  |  | - | 0.0% |  | **0 fewer per 1,000** (from 0 fewer to --) |  |  |
| **Spatiotemporal RL - Left stance phase (s)** | | | | | | | | | | | | |
| 2 | non-randomised studies | not serious | not serious | not serious | not serious | very strong association all plausible residual confounding would reduce the demonstrated effect dose response gradient | 88 cases 37 controls | | **RR 0.02** (0.01 to 0.03) | - | ⨁⨁⨁⨁ High |  |
|  |  |  |  |  |  |  | - | 0.0% |  | **0 fewer per 1,000** (from 0 fewer to 0 fewer) |  |  |
| **Spatiotemporal RL - Right Swing phase (%)** | | | | | | | | | | | | |
| 2 | non-randomised studies | not serious | not serious | not serious | serious | strong association all plausible residual confounding would reduce the demonstrated effect dose response gradient | 40 cases 40 controls | | **RR -2.05** (-2.40 to -1.69) | - | ⨁⨁⨁⨁ High |  |
|  |  |  |  |  |  |  | - | 0.0% |  | **0 fewer per 1,000** (from 0 fewer to 0 fewer) |  |  |
| **Spatiotemporal RL - Left Swing phase (%)** | | | | | | | | | | | | |
| 2 | non-randomised studies | not serious | not serious | not serious | serious | very strong association all plausible residual confounding would reduce the demonstrated effect dose response gradient | 40 cases 40 controls | | **RR -0.85** (-1.18 to -0.52) | - | ⨁⨁⨁⨁ High |  |
|  |  |  |  |  |  |  | - | 0.0% |  | **0 fewer per 1,000** (from 0 fewer to 0 fewer) |  |  |
| **Spatiotemporal RL - Right Swing phase (s)** | | | | | | | | | | | | |
| 1 | non-randomised studies | not serious | not serious | not serious | serious | all plausible residual confounding would reduce the demonstrated effect dose response gradient | 20 cases 20 controls | | **RR 0.00** (-0.02 to 0.02) | - | ⨁⨁⨁◯ Moderate |  |
|  |  |  |  |  |  |  | - | 0.0% |  |  |  |  |
| **Spatiotemporal RL - Left Swing phase (s)** | | | | | | | | | | | | |
| 1 | non-randomised studies | not serious | not serious | not serious | serious | all plausible residual confounding would reduce the demonstrated effect dose response gradient | 20 cases 20 controls | | **RR 0.02** (0.00 to 0.04) | - | ⨁⨁⨁◯ Moderate |  |
|  |  |  |  |  |  |  | - | 0.0% |  | **0 fewer per 1,000** (from 0 fewer to --) |  |  |
| **Spatiotemporal RL - Right Step time (s)** | | | | | | | | | | | | |
| 3 | non-randomised studies | not serious | not serious | not serious | serious | all plausible residual confounding would reduce the demonstrated effect dose response gradient | 48 cases 49 controls | | **RR -0.01** (-0.02 to 0.01) | - | ⨁⨁⨁◯ Moderate |  |
|  |  |  |  |  |  |  | - | 0.0% |  | **0 fewer per 1,000** (from 0 fewer to 0 fewer) |  |  |
| **Spatiotemporal RL - Left Step time (s)** | | | | | | | | | | | | |
| 3 | non-randomised studies | not serious | serious | not serious | serious | strong association all plausible residual confounding would reduce the demonstrated effect dose response gradient | 48 cases 49 controls | | **RR 0.01** (-0.03 to 0.04) | - | ⨁⨁⨁◯ Moderate |  |
|  |  |  |  |  |  |  | - | 0.0% |  | **0 fewer per 1,000** (from 0 fewer to 0 fewer) |  |  |
| **Spatiotemporal RL - Right Stride time (s)** | | | | | | | | | | | | |
| 3 | non-randomised studies | not serious | not serious | not serious | serious | all plausible residual confounding would reduce the demonstrated effect dose response gradient | 47 cases 48 controls | | **RR 0.02** (-0.01 to 0.05) | - | ⨁⨁⨁◯ Moderate |  |
|  |  |  |  |  |  |  | - | 0.0% |  | **0 fewer per 1,000** (from 0 fewer to 0 fewer) |  |  |
| **Spatiotemporal RL - Left Stride time (s)** | | | | | | | | | | | | |
| 3 | non-randomised studies | not serious | not serious | not serious | serious | strong association all plausible residual confounding would reduce the demonstrated effect dose response gradient | 47 cases 48 controls | | **RR 0.01** (-0.03 to 0.05) | - | ⨁⨁⨁⨁ High |  |
|  |  |  |  |  |  |  | - | 0.0% |  | **0 fewer per 1,000** (from 0 fewer to 0 fewer) |  |  |
| **Standard - Stride Length** | | | | | | | | | | | | |
| 7 | non-randomised studies | not serious | very serious | not serious | serious | all plausible residual confounding would reduce the demonstrated effect dose response gradient | 200 cases 137 controls | | **RR -0.15** (-1.03 to 0.73) | - | ⨁◯◯◯ Very low |  |
|  |  |  |  |  |  |  | - | 0.0% |  | **0 fewer per 1,000** (from 0 fewer to 0 fewer) |  |  |
| **Standard - Stride time** | | | | | | | | | | | | |
| 3 | non-randomised studies | not serious | very serious | not serious | very serious | all plausible residual confounding would reduce the demonstrated effect dose response gradient | 63 cases 58 controls | | **RR -1.45** (-3.77 to 0.86) | - | ⨁◯◯◯ Very low |  |
|  |  |  |  |  |  |  | - | 0.0% |  | **0 fewer per 1,000** (from 0 fewer to 0 fewer) |  |  |
| **Standard - Step width** | | | | | | | | | | | | |
| 5 | non-randomised studies | not serious | very serious | not serious | serious | all plausible residual confounding would reduce the demonstrated effect dose response gradient | 136 cases 80 controls | | **RR 0.27** (-0.55 to 1.09) | - | ⨁◯◯◯ Very low |  |
|  |  |  |  |  |  |  | - | 0.0% |  | **0 fewer per 1,000** (from 0 fewer to 0 fewer) |  |  |
| **Standard - Stance time** | | | | | | | | | | | | |
| 6 | non-randomised studies | not serious | very serious | not serious | serious | all plausible residual confounding would reduce the demonstrated effect dose response gradient | 114 cases 97 controls | | **RR 0.07** (-0.74 to 0.88) | - | ⨁◯◯◯ Very low |  |
|  |  |  |  |  |  |  | - | 0.0% |  | **0 fewer per 1,000** (from 0 fewer to 0 fewer) |  |  |
| **Standard - Swing time** | | | | | | | | | | | | |
| 3 | non-randomised studies | not serious | not serious | not serious | serious | all plausible residual confounding would reduce the demonstrated effect dose response gradient | 47 cases 42 controls | | **RR 0.13** (-0.39 to 0.65) | - | ⨁⨁⨁◯ Moderate |  |
|  |  |  |  |  |  |  | - | 0.0% |  | **0 fewer per 1,000** (from 0 fewer to 0 fewer) |  |  |
| **Standard RL - Right Cadence** | | | | | | | | | | | | |
| 5 | non-randomised studies | not serious | not serious | not serious | serious | all plausible residual confounding would reduce the demonstrated effect dose response gradient | 59 cases 71 controls | | **RR -0.57** (-1.28 to 0.14) | - | ⨁⨁⨁◯ Moderate |  |
|  |  |  |  |  |  |  | - | 0.0% |  | **0 fewer per 1,000** (from 0 fewer to 0 fewer) |  |  |
| **Standard RL - Left Cadence** | | | | | | | | | | | | |
| 4 | non-randomised studies | not serious | not serious | not serious | serious | all plausible residual confounding would reduce the demonstrated effect dose response gradient | 46 cases 58 controls | | **RR -0.21** (-0.72 to 0.29) | - | ⨁⨁⨁◯ Moderate |  |
|  |  |  |  |  |  |  | - | 0.0% |  | **0 fewer per 1,000** (from 0 fewer to 0 fewer) |  |  |
| **Standard RL - Right Speed** | | | | | | | | | | | | |
| 3 | non-randomised studies | not serious | not serious | not serious | serious | all plausible residual confounding would reduce the demonstrated effect dose response gradient | 40 cases 41 controls | | **RR -0.16** (-0.56 to 0.24) | - | ⨁⨁⨁◯ Moderate |  |
|  |  |  |  |  |  |  | - | 0.0% |  | **0 fewer per 1,000** (from 0 fewer to 0 fewer) |  |  |
| **Standard RL - Left Speed** | | | | | | | | | | | | |
| 2 | non-randomised studies | not serious | not serious | not serious | serious | all plausible residual confounding would reduce the demonstrated effect dose response gradient | 27 cases 28 controls | | **RR -0.03** (-0.56 to 0.50) | - | ⨁⨁⨁◯ Moderate |  |
|  |  |  |  |  |  |  | - | 0.0% |  | **0 fewer per 1,000** (from 0 fewer to 0 fewer) |  |  |
| **Standard RL - Right Stride Length** | | | | | | | | | | | | |
| 4 | non-randomised studies | not serious | serious | not serious | serious | all plausible residual confounding would reduce the demonstrated effect dose response gradient | 68 cases 81 controls | | **RR -0.44** (-1.03 to 0.15) | - | ⨁⨁◯◯ Low |  |
|  |  |  |  |  |  |  | - | 0.0% |  | **0 fewer per 1,000** (from 0 fewer to 0 fewer) |  |  |
| **Standard RL - Left Stride Length** | | | | | | | | | | | | |
| 4 | non-randomised studies | not serious | serious | not serious | serious | all plausible residual confounding would reduce the demonstrated effect dose response gradient | 68 cases 81 controls | | **RR -0.32** (-0.90 to 0.26) | - | ⨁⨁◯◯ Low |  |
|  |  |  |  |  |  |  | - | 0.0% |  | **0 fewer per 1,000** (from 0 fewer to 0 fewer) |  |  |
| **Standard RL - Left step length** | | | | | | | | | | | | |
| 6 | non-randomised studies | not serious | very serious | not serious | serious | all plausible residual confounding would reduce the demonstrated effect dose response gradient | 153 cases 104 controls | | **RR 0.43** (-0.51 to 1.36) | - | ⨁◯◯◯ Very low |  |
|  |  |  |  |  |  |  | - | 0.0% |  | **0 fewer per 1,000** (from 0 fewer to 0 fewer) |  |  |
| **Standard RL - Right Step length** | | | | | | | | | | | | |
| 8 | non-randomised studies | not serious | very serious | not serious | serious | all plausible residual confounding would reduce the demonstrated effect dose response gradient | 186 cases 137 controls | | **RR 0.43** (-0.17 to 1.02) | - | ⨁◯◯◯ Very low |  |
|  |  |  |  |  |  |  | - | 0.0% |  | **0 fewer per 1,000** (from 0 fewer to 0 fewer) |  |  |
| **Standard RL - Left Double support** | | | | | | | | | | | | |
| 3 | non-randomised studies | not serious | not serious | not serious | serious | all plausible residual confounding would reduce the demonstrated effect dose response gradient | 47 cases 48 controls | | **RR 0.33** (-0.30 to 0.96) | - | ⨁⨁⨁◯ Moderate |  |
|  |  |  |  |  |  |  | - | 0.0% |  | **0 fewer per 1,000** (from 0 fewer to 0 fewer) |  |  |
| **Standard RL - Right Double support** | | | | | | | | | | | | |
| 3 | non-randomised studies | not serious | not serious | not serious | serious | all plausible residual confounding would reduce the demonstrated effect dose response gradient | 47 cases 48 controls | | **RR 0.02** (-0.51 to 0.56) | - | ⨁⨁⨁◯ Moderate |  |
|  |  |  |  |  |  |  | - | 0.0% |  | **0 fewer per 1,000** (from 0 fewer to 0 fewer) |  |  |
| **Standard RL - Right Single support** | | | | | | | | | | | | |
| 4 | non-randomised studies | not serious | not serious | not serious | serious | all plausible residual confounding would reduce the demonstrated effect dose response gradient | 67 cases 68 controls | | **RR 0.16** (-0.18 to 0.50) | - | ⨁⨁⨁◯ Moderate |  |
|  |  |  |  |  |  |  | - | 0.0% |  | **0 fewer per 1,000** (from 0 fewer to 0 fewer) |  |  |
| **Standard RL - Left Single support** | | | | | | | | | | | | |
| 4 | non-randomised studies | not serious | very serious | not serious | serious | all plausible residual confounding would reduce the demonstrated effect dose response gradient | 67 cases 68 controls | | **RR -0.48** (-0.89 to -0.08) | - | ⨁◯◯◯ Very low |  |
|  |  |  |  |  |  |  | - | 0.0% |  | **0 fewer per 1,000** (from 0 fewer to 0 fewer) |  |  |

**CI:** confidence interval; **RR:** risk ratio
