# Supplementary material for: Effects of spinal deformities on lower limb kinematics during walking: a systematic review and meta-analysis
Source: Sci Rep. 2025 Feb 7;15:4608. doi: 10.1038/s41598-025-88886-5 (PMC11806027; doi:10.1038/s41598-025-88886-5)
Supplement: Supplementary file 2 — Supplementary Material 1 [file 41598_2025_88886_MOESM2_ESM.docx]

ASD compared to Control

| **Certainty assessment** | | | | | | | **№ of patients** | | **Effect** | | **Certainty** | **Importance** |
| --- | --- | --- | --- | --- | --- | --- | --- | --- | --- | --- | --- | --- |
| **№ of studies** | **Study design** | **Risk of bias** | **Inconsistency** | **Indirectness** | **Imprecision** | **Other considerations** | **ASD** | **Control** | **Relative (95% CI)** | **Absolute (95% CI)** |  |  |
| **Spatiotemporal - Speed (m/s)** | | | | | | | | | | | | |
| 1 | non-randomised studies | not serious | not serious | not serious | not serious | strong association all plausible residual confounding would reduce the demonstrated effect dose response gradient | 52 cases 46 controls | | **RR -0.14** (-0.20 to -0.08) | - | ⨁⨁⨁⨁ High |  |
|  |  |  |  |  |  |  | - | 0.0% |  | **0 fewer per 1,000** (from 0 fewer to 0 fewer) |  |  |
| **Spatiotemporal - Step width** | | | | | | | | | | | | |
| 1 | non-randomised studies | not serious | not serious | not serious | not serious | all plausible residual confounding would reduce the demonstrated effect dose response gradient | 52 cases 46 controls | | **RR 0.01** (-0.01 to 0.03) | - | ⨁⨁⨁⨁ High |  |
|  |  |  |  |  |  |  | - | 0.0% |  | **0 fewer per 1,000** (from 0 fewer to 0 fewer) |  |  |
| **Spatiotemporal - Stride Left (cm)** | | | | | | | | | | | | |
| 1 | non-randomised studies | not serious | not serious | not serious | not serious | very strong association all plausible residual confounding would reduce the demonstrated effect dose response gradient | 33 cases 33 controls | | **RR -18.10** (-25.31 to -10.89) | - | ⨁⨁⨁⨁ High |  |
|  |  |  |  |  |  |  | - | 0.0% |  | **0 fewer per 1,000** (from 0 fewer to 0 fewer) |  |  |
| **Spatiotemporal - Stride RT-LF difference (cm)** | | | | | | | | | | | | |
| 1 | non-randomised studies | not serious | not serious | not serious | not serious | very strong association all plausible residual confounding would reduce the demonstrated effect dose response gradient | 33 cases 33 controls | | **RR 2.20** (1.29 to 3.11) | - | ⨁⨁⨁⨁ High |  |
|  |  |  |  |  |  |  | - | 0.0% |  | **0 fewer per 1,000** (from 0 fewer to 0 fewer) |  |  |
| **Spatiotemporal - Stance phace (%)** | | | | | | | | | | | | |
| 1 | non-randomised studies | not serious | not serious | not serious | not serious | all plausible residual confounding would reduce the demonstrated effect dose response gradient | 33 cases 33 controls | | **RR 0.10** (-1.25 to 1.45) | - | ⨁⨁⨁⨁ High |  |
|  |  |  |  |  |  |  | - | 0.0% |  | **0 fewer per 1,000** (from 0 fewer to 0 fewer) |  |  |
| **Spatiotemporal - Swing phace (%)** | | | | | | | | | | | | |
| 1 | non-randomised studies | not serious | not serious | not serious | not serious | all plausible residual confounding would reduce the demonstrated effect dose response gradient | 33 cases 33 controls | | **RR 0.10** (-1.25 to 1.45) | - | ⨁⨁⨁⨁ High |  |
|  |  |  |  |  |  |  | - | 0.0% |  | **0 fewer per 1,000** (from 0 fewer to 0 fewer) |  |  |
| **Spatiotemporal - Foot Off (%)** | | | | | | | | | | | | |
| 1 | non-randomised studies | not serious | not serious | not serious | not serious | strong association all plausible residual confounding would reduce the demonstrated effect dose response gradient | 52 cases 63 controls | | **RR 1.50** (0.05 to 2.95) | - | ⨁⨁⨁⨁ High |  |
|  |  |  |  |  |  |  | - | 0.0% |  | **0 fewer per 1,000** (from 0 fewer to 0 fewer) |  |  |
| **Spatiotemporal - Single Suport (s)** | | | | | | | | | | | | |
| 1 | non-randomised studies | not serious | not serious | not serious | not serious | all plausible residual confounding would reduce the demonstrated effect dose response gradient | 52 cases 63 controls | | **RR 0.03** (0.00 to 0.06) | - | ⨁⨁⨁⨁ High |  |
|  |  |  |  |  |  |  | - | 0.0% |  | **0 fewer per 1,000** (from 0 fewer to --) |  |  |
| **Spatiotemporal - Step Length (m)** | | | | | | | | | | | | |
| 2 | non-randomised studies | not serious | not serious | not serious | not serious | very strong association all plausible residual confounding would reduce the demonstrated effect dose response gradient | 176 cases 110 controls | | **RR -0.10** (-0.12 to -0.08) | - | ⨁⨁⨁⨁ High |  |
|  |  |  |  |  |  |  | - | 0.0% |  | **0 fewer per 1,000** (from 0 fewer to 0 fewer) |  |  |
| **Angle - balanced ASD Knee Extention (°)** | | | | | | | | | | | | |
| 1 | non-randomised studies | not serious | not serious | not serious | not serious | all plausible residual confounding would reduce the demonstrated effect dose response gradient | 69 cases 62 controls | | **RR 1.40** (-1.18 to 3.98) | - | ⨁⨁⨁⨁ High |  |
|  |  |  |  |  |  |  | - | 0.0% |  | **0 fewer per 1,000** (from 0 fewer to 0 fewer) |  |  |
| **Angle - Unbalanced ASD Knee Extention (°)** | | | | | | | | | | | | |
| 1 | non-randomised studies | not serious | not serious | not serious | not serious | very strong association all plausible residual confounding would reduce the demonstrated effect dose response gradient | 69 cases 62 controls | | **RR 12.30** (9.04 to 15.56) | - | ⨁⨁⨁⨁ High |  |
|  |  |  |  |  |  |  | - | 0.0% |  | **0 fewer per 1,000** (from 0 fewer to 0 fewer) |  |  |
| **Angle - Hip Rotation ROM (°)** | | | | | | | | | | | | |
| 1 | non-randomised studies | not serious | not serious | not serious | not serious | all plausible residual confounding would reduce the demonstrated effect dose response gradient | 52 cases 63 controls | | **RR 2.6** (-4.0 to 9.2) | - | ⨁⨁⨁⨁ High |  |
|  |  |  |  |  |  |  | - | 0.0% |  | **0 fewer per 1,000** (from 0 fewer to 0 fewer) |  |  |
| **Angle - Mean Hip sagittal angle (°)** | | | | | | | | | | | | |
| 2 | non-randomised studies | not serious | serious | not serious | not serious | all plausible residual confounding would reduce the demonstrated effect dose response gradient | 176 cases 110 controls | | **RR -0.33** (-5.03 to 4.37) | - | ⨁⨁⨁◯ Moderate |  |
|  |  |  |  |  |  |  | - | 0.0% |  | **0 fewer per 1,000** (from 0 fewer to 0 fewer) |  |  |
| **Angle - Hip sagital ROM (°)** | | | | | | | | | | | | |
| 2 | non-randomised studies | not serious | not serious | not serious | not serious | very strong association all plausible residual confounding would reduce the demonstrated effect dose response gradient | 176 cases 110 controls | | **RR -3.30** (-4.74 to -1.86) | - | ⨁⨁⨁⨁ High |  |
|  |  |  |  |  |  |  | - | 0.0% |  | **0 fewer per 1,000** (from 0 fewer to 0 fewer) |  |  |
| **Angle - Mean Hip Abduction (°)** | | | | | | | | | | | | |
| 1 | non-randomised studies | not serious | not serious | not serious | not serious | all plausible residual confounding would reduce the demonstrated effect dose response gradient | 52 cases 63 controls | | **RR 0.00** (-1.38 to 1.38) | - | ⨁⨁⨁⨁ High |  |
|  |  |  |  |  |  |  | - | 0.0% |  |  |  |  |
| **Angle - Knee sagital ROM (°)** | | | | | | | | | | | | |
| 2 | non-randomised studies | not serious | not serious | not serious | not serious | all plausible residual confounding would reduce the demonstrated effect dose response gradient | 176 cases 110 controls | | **RR 0.94** (-0.80 to 2.68) | - | ⨁⨁⨁⨁ High |  |
|  |  |  |  |  |  |  | - | 0.0% |  | **0 fewer per 1,000** (from 0 fewer to 0 fewer) |  |  |
| **Angle - Mean knee sagittal angle (°)** | | | | | | | | | | | | |
| 1 | non-randomised studies | not serious | not serious | not serious | not serious | very strong association all plausible residual confounding would reduce the demonstrated effect dose response gradient | 124 cases 47 controls | | **RR -5.10** (-7.48 to -2.72) | - | ⨁⨁⨁⨁ High |  |
|  |  |  |  |  |  |  | - | 0.0% |  | **0 fewer per 1,000** (from 0 fewer to 0 fewer) |  |  |
| **Standard spatiotemporal - Velocity (m/s)** | | | | | | | | | | | | |
| 2 | non-randomised studies | not serious | serious | not serious | not serious | very strong association all plausible residual confounding would reduce the demonstrated effect dose response gradient | 85 cases 96 controls | | **RR -1.07** (-1.67 to -0.47) | - | ⨁⨁⨁⨁ High |  |
|  |  |  |  |  |  |  | - | 0.0% |  | **0 fewer per 1,000** (from 0 fewer to 0 fewer) |  |  |
| **Standard spatiotemporal - Double Support (s)** | | | | | | | | | | | | |
| 3 | non-randomised studies | not serious | not serious | not serious | not serious | very strong association all plausible residual confounding would reduce the demonstrated effect dose response gradient | 209 cases 143 controls | | **RR 0.51** (0.26 to 0.76) | - | ⨁⨁⨁⨁ High |  |
|  |  |  |  |  |  |  | - | 0.0% |  | **0 fewer per 1,000** (from 0 fewer to 0 fewer) |  |  |
| **Standard spatiotemporal - Cadence(steps/s)** | | | | | | | | | | | | |
| 4 | non-randomised studies | not serious | not serious | not serious | not serious | very strong association all plausible residual confounding would reduce the demonstrated effect dose response gradient | 261 cases 189 controls | | **RR -0.82** (-1.06 to -0.58) | - | ⨁⨁⨁⨁ High |  |
|  |  |  |  |  |  |  | - | 0.0% |  | **0 fewer per 1,000** (from 0 fewer to 0 fewer) |  |  |
| **Standard spatiotemporal - Stride length Right (cm)** | | | | | | | | | | | | |
| 2 | non-randomised studies | not serious | not serious | not serious | not serious | very strong association all plausible residual confounding would reduce the demonstrated effect dose response gradient | 85 cases 79 controls | | **RR -0.95** (-1.28 to -0.63) | - | ⨁⨁⨁⨁ High |  |
|  |  |  |  |  |  |  | - | 0.0% |  | **0 fewer per 1,000** (from 0 fewer to 0 fewer) |  |  |

**CI:** confidence interval; **RR:** risk ratio
