# Supplementary material for: Effects of spinal deformities on lower limb kinematics during walking: a systematic review and meta-analysis
Source: Sci Rep. 2025 Feb 7;15:4608. doi: 10.1038/s41598-025-88886-5 (PMC11806027; doi:10.1038/s41598-025-88886-5)
Supplement: Supplementary file 3 — Supplementary Material 1 [file 41598_2025_88886_MOESM3_ESM.docx]

Sagital malalignment compared to Control

| **Certainty assessment** | | | | | | | **№ of patients** | | **Effect** | | **Certainty** | **Importance** |
| --- | --- | --- | --- | --- | --- | --- | --- | --- | --- | --- | --- | --- |
| **№ of studies** | **Study design** | **Risk of bias** | **Inconsistency** | **Indirectness** | **Imprecision** | **Other considerations** | **Sagital malalignment** | **Control** | **Relative (95% CI)** | **Absolute (95% CI)** |  |  |
| **Spatiotemporal - Step length right (cm)** | | | | | | | | | | | | |
| 1 | non-randomised studies | not serious | not serious | not serious | serious | strong association all plausible residual confounding would reduce the demonstrated effect dose response gradient | 12 cases 13 controls | | **RR -4.43** (-6.87 to -1.99) | - | ⨁⨁⨁⨁ High |  |
|  |  |  |  |  |  |  | - | 0.0% |  | **0 fewer per 1,000** (from 0 fewer to 0 fewer) |  |  |
| **Spatiotemporal - Step length left (cm)** | | | | | | | | | | | | |
| 1 | non-randomised studies | not serious | not serious | not serious | serious | strong association all plausible residual confounding would reduce the demonstrated effect dose response gradient | 12 cases 13 controls | | **RR -2.87** (-5.24 to -0.50) | - | ⨁⨁⨁⨁ High |  |
|  |  |  |  |  |  |  | - | 0.0% |  | **0 fewer per 1,000** (from 0 fewer to 0 fewer) |  |  |
| **Spatiotemporal - Stride length Left (cm)** | | | | | | | | | | | | |
| 1 | non-randomised studies | not serious | not serious | not serious | serious | very strong association all plausible residual confounding would reduce the demonstrated effect dose response gradient | 12 cases 13 controls | | **RR -24.1** (-29.6 to -18.6) | - | ⨁⨁⨁⨁ High |  |
|  |  |  |  |  |  |  | - | 0.0% |  | **0 fewer per 1,000** (from 0 fewer to 0 fewer) |  |  |
| **Spatiotemporal - Stride length Right (cm)** | | | | | | | | | | | | |
| 2 | non-randomised studies | not serious | very serious | not serious | serious | all plausible residual confounding would reduce the demonstrated effect dose response gradient | 21 cases 23 controls | | **RR -17.27** (-37.16 to 2.62) | - | ⨁◯◯◯ Very low |  |
|  |  |  |  |  |  |  | - | 0.0% |  | **0 fewer per 1,000** (from 0 fewer to 0 fewer) |  |  |
| **Spatiotemporal - Stance phace (%)** | | | | | | | | | | | | |
| 1 | non-randomised studies | not serious | not serious | not serious | serious | strong association all plausible residual confounding would reduce the demonstrated effect dose response gradient | 33 cases 36 controls | | **RR 4.70** (1.88 to 7.52) | - | ⨁⨁⨁⨁ High |  |
|  |  |  |  |  |  |  | - | 0.0% |  | **0 fewer per 1,000** (from 0 fewer to 0 fewer) |  |  |
| **Spatiotemporal - Swing phace (%)** | | | | | | | | | | | | |
| 1 | non-randomised studies | not serious | not serious | not serious | serious | strong association all plausible residual confounding would reduce the demonstrated effect dose response gradient | 21 cases 23 controls | | **RR -4.60** (-7.42 to -1.78) | - | ⨁⨁⨁⨁ High |  |
|  |  |  |  |  |  |  | - | 0.0% |  | **0 fewer per 1,000** (from 0 fewer to 0 fewer) |  |  |
| **Spatiotemporal - Right single support (%)** | | | | | | | | | | | | |
| 1 | non-randomised studies | not serious | not serious | not serious | serious | all plausible residual confounding would reduce the demonstrated effect dose response gradient | 12 cases 13 controls | | **RR -1.29** (-2.72 to 0.14) | - | ⨁⨁⨁◯ Moderate |  |
|  |  |  |  |  |  |  | - | 0.0% |  | **0 fewer per 1,000** (from 0 fewer to 0 fewer) |  |  |
| **Spatiotemporal - Left single support (%)** | | | | | | | | | | | | |
| 1 | non-randomised studies | not serious | not serious | not serious | serious | all plausible residual confounding would reduce the demonstrated effect dose response gradient | 12 cases 13 controls | | **RR 0.00** (-1.41 to 1.41) | - | ⨁⨁⨁◯ Moderate |  |
|  |  |  |  |  |  |  | - | 0.0% |  |  |  |  |
| **Spatiotemporal - Single Support (s)** | | | | | | | | | | | | |
| 1 | non-randomised studies | not serious | not serious | not serious | serious | all plausible residual confounding would reduce the demonstrated effect dose response gradient | 30 cases 36 controls | | not estimable | - | ⨁⨁⨁◯ Moderate |  |
|  |  |  |  |  |  |  | - | 0.0% |  |  |  |  |
| **Spatiotemporal - Step Time (s)** | | | | | | | | | | | | |
| 1 | non-randomised studies | not serious | not serious | not serious | serious | very strong association all plausible residual confounding would reduce the demonstrated effect dose response gradient | 30 cases 36 controls | | **RR 0.05** (-0.05 to 0.15) | - | ⨁⨁⨁⨁ High |  |
|  |  |  |  |  |  |  | - | 0.0% |  | **0 fewer per 1,000** (from 0 fewer to 0 fewer) |  |  |
| **Spatiotemporal - Foot Off (%)** | | | | | | | | | | | | |
| 1 | non-randomised studies | not serious | not serious | not serious | serious | strong association all plausible residual confounding would reduce the demonstrated effect dose response gradient | 30 cases 36 controls | | **RR 1.28** (0.10 to 2.45) | - | ⨁⨁⨁⨁ High |  |
|  |  |  |  |  |  |  | - | 0.0% |  | **0 fewer per 1,000** (from 0 fewer to 0 fewer) |  |  |
| **Spatiotemporal - Step lengh/ height (%)** | | | | | | | | | | | | |
| 1 | non-randomised studies | not serious | not serious | not serious | serious | all plausible residual confounding would reduce the demonstrated effect dose response gradient | 14 cases 14 controls | | **RR -0.03** (-0.06 to 0.00) | - | ⨁⨁⨁◯ Moderate |  |
|  |  |  |  |  |  |  | - | 0.0% |  | **0 fewer per 1,000** (from -- to 0 fewer) |  |  |
| **Spatiotemporal - Step width (cm)** | | | | | | | | | | | | |
| 2 | non-randomised studies | not serious | not serious | not serious | serious | all plausible residual confounding would reduce the demonstrated effect dose response gradient | 33 cases 36 controls | | **RR 1.40** (-0.23 to 3.04) | - | ⨁⨁⨁◯ Moderate |  |
|  |  |  |  |  |  |  | - | 0.0% |  | **0 fewer per 1,000** (from 0 fewer to 0 fewer) |  |  |
| **Spatiotemporal - Cycle time (s)** | | | | | | | | | | | | |
| 1 | non-randomised studies | not serious | not serious | not serious | serious | strong association all plausible residual confounding would reduce the demonstrated effect dose response gradient | 20 cases 12 controls | | **RR 0.08** (0.00 to 0.16) | - | ⨁⨁⨁⨁ High |  |
|  |  |  |  |  |  |  | - | 0.0% |  | **0 fewer per 1,000** (from 0 fewer to --) |  |  |
| **Angle - Hip sagital ROM (°) in stance** | | | | | | | | | | | | |
| 1 | non-randomised studies | not serious | not serious | not serious | not serious | all plausible residual confounding would reduce the demonstrated effect dose response gradient | 91 cases 120 controls | | **RR -4.08** (-8.29 to 0.13) | - | ⨁⨁⨁⨁ High |  |
|  |  |  |  |  |  |  | - | 0.0% |  | **0 fewer per 1,000** (from 0 fewer to 0 fewer) |  |  |
| **Angle - Hip maximum extention in stance (°)** | | | | | | | | | | | | |
| 1 | non-randomised studies | not serious | not serious | not serious | not serious | all plausible residual confounding would reduce the demonstrated effect dose response gradient | 91 cases 120 controls | | **RR -0.43** (-7.09 to 6.23) | - | ⨁⨁⨁⨁ High |  |
|  |  |  |  |  |  |  | - | 0.0% |  | **0 fewer per 1,000** (from 0 fewer to 0 fewer) |  |  |
| **Angle - Knee maximum flexion in stance (°)** | | | | | | | | | | | | |
| 1 | non-randomised studies | not serious | not serious | not serious | not serious | all plausible residual confounding would reduce the demonstrated effect dose response gradient | 91 cases 120 controls | | **RR 0.4** (-2.3 to 3.1) | - | ⨁⨁⨁⨁ High |  |
|  |  |  |  |  |  |  | - | 0.0% |  | **0 fewer per 1,000** (from 0 fewer to 0 fewer) |  |  |
| **Angle - Knee maximum extention in stance (°)** | | | | | | | | | | | | |
| 1 | non-randomised studies | not serious | not serious | not serious | not serious | all plausible residual confounding would reduce the demonstrated effect dose response gradient | 91 cases 120 controls | | **RR 3.06** (-0.86 to 6.98) | - | ⨁⨁⨁⨁ High |  |
|  |  |  |  |  |  |  | - | 0.0% |  | **0 fewer per 1,000** (from 0 fewer to 0 fewer) |  |  |
| **Angle - Knee maximum extention at initial contact (°)** | | | | | | | | | | | | |
| 1 | non-randomised studies | not serious | not serious | not serious | not serious | very strong association all plausible residual confounding would reduce the demonstrated effect dose response gradient | 91 cases 120 controls | | **RR 3.98** (1.05 to 6.91) | - | ⨁⨁⨁⨁ High |  |
|  |  |  |  |  |  |  | - | 0.0% |  | **0 fewer per 1,000** (from 0 fewer to 0 fewer) |  |  |
| **Angle - Knee mean sagittal angle (°)** | | | | | | | | | | | | |
| 1 | non-randomised studies | not serious | not serious | not serious | not serious | all plausible residual confounding would reduce the demonstrated effect dose response gradient | 91 cases 120 controls | | **RR -9.75** (-28.86 to 9.36) | - | ⨁⨁⨁⨁ High |  |
|  |  |  |  |  |  |  | - | 0.0% |  | **0 fewer per 1,000** (from 0 fewer to 0 fewer) |  |  |
| **Angle - Ankle peak plantarflexion in stance (°)** | | | | | | | | | | | | |
| 1 | non-randomised studies | not serious | not serious | not serious | not serious | very strong association all plausible residual confounding would reduce the demonstrated effect dose response gradient | 91 cases 120 controls | | **RR -15.29** (-17.33 to -13.25) | - | ⨁⨁⨁⨁ High |  |
|  |  |  |  |  |  |  | - | 0.0% |  | **0 fewer per 1,000** (from 0 fewer to 0 fewer) |  |  |
| **Angle - Ankle peak dorsiflexion in swing (°)** | | | | | | | | | | | | |
| 1 | non-randomised studies | not serious | not serious | not serious | not serious | all plausible residual confounding would reduce the demonstrated effect dose response gradient | 91 cases 120 controls | | **RR 0.23** (-1.40 to 1.86) | - | ⨁⨁⨁⨁ High |  |
|  |  |  |  |  |  |  | - | 0.0% |  | **0 fewer per 1,000** (from 0 fewer to 0 fewer) |  |  |
| **Angle - Foot mean progression in stance (°)** | | | | | | | | | | | | |
| 1 | non-randomised studies | not serious | serious | not serious | not serious | all plausible residual confounding would reduce the demonstrated effect dose response gradient | 91 cases 120 controls | | **RR -1.11** (-4.70 to 2.49) | - | ⨁⨁⨁◯ Moderate |  |
|  |  |  |  |  |  |  | - | 0.0% |  | **0 fewer per 1,000** (from 0 fewer to 0 fewer) |  |  |
| **Angle - Foot progression ROM in stance (°)** | | | | | | | | | | | | |
| 1 | non-randomised studies | not serious | not serious | not serious | not serious | all plausible residual confounding would reduce the demonstrated effect dose response gradient | 91 cases 120 controls | | **RR 0.14** (-1.33 to 1.61) | - | ⨁⨁⨁⨁ High |  |
|  |  |  |  |  |  |  | - | 0.0% |  | **0 fewer per 1,000** (from 0 fewer to 0 fewer) |  |  |
| **Angle- no meta - Knee maximum flexion in swing (°)** | | | | | | | | | | | | |
| 2 | non-randomised studies | not serious | not serious | serious | not serious | all plausible residual confounding would reduce the demonstrated effect dose response gradient | 91 cases 120 controls | | not pooled | - | ⨁⨁⨁◯ Moderate |  |
|  |  |  |  |  |  |  | - | 0.0% |  | not pooled |  |  |
| **Angle- no meta - Ankle Max dorsiflexion in stance (°)** | | | | | | | | | | | | |
| 2 | non-randomised studies | not serious | not serious | serious | not serious | all plausible residual confounding would reduce the demonstrated effect dose response gradient | 121 cases 150 controls | | not pooled | - | ⨁⨁⨁◯ Moderate |  |
|  |  |  |  |  |  |  | - | 0.0% |  | not pooled |  |  |
| **Angle- no meta - Ankle maximum plantarflexion (°)** | | | | | | | | | | | | |
| 1 | non-randomised studies | not serious | not serious | serious | serious | all plausible residual confounding would reduce the demonstrated effect dose response gradient | 121 cases 150 controls | | not estimable | - | ⨁⨁◯◯ Low |  |
|  |  |  |  |  |  |  | - | 0.0% |  |  |  |  |
| **Angle- no meta - Knee maximum extention (°)** | | | | | | | | | | | | |
| 1 | non-randomised studies | not serious | not serious | serious | serious | all plausible residual confounding would reduce the demonstrated effect dose response gradient | 30 cases 30 controls | | not estimable | - | ⨁⨁◯◯ Low |  |
|  |  |  |  |  |  |  | - | 0.0% |  |  |  |  |
| **Angle- no meta - Hip peak flexion angle in loading response (°)** | | | | | | | | | | | | |
| 1 | non-randomised studies | not serious | not serious | not serious | serious | all plausible residual confounding would reduce the demonstrated effect dose response gradient | 30 cases 30 controls | | **RR -3.30** (-6.66 to 0.06) | - | ⨁⨁⨁◯ Moderate |  |
|  |  |  |  |  |  |  | - | 0.0% |  | **0 fewer per 1,000** (from 0 fewer to 0 fewer) |  |  |
| **Angle- no meta - Hip peak extention angle in pre-swing (°)** | | | | | | | | | | | | |
| 2 | non-randomised studies | not serious | not serious | serious | serious | strong association all plausible residual confounding would reduce the demonstrated effect dose response gradient | 20 cases 12 controls | | not pooled | - | ⨁⨁⨁◯ Moderate |  |
|  |  |  |  |  |  |  | - | 0.0% |  | not pooled |  |  |
| **Angle- no meta - Knee peak extention angle in single support (°)** | | | | | | | | | | | | |
| 1 | non-randomised studies | not serious | not serious | not serious | serious | all plausible residual confounding would reduce the demonstrated effect dose response gradient | 20 cases 12 controls | | **RR 2.30** (-1.83 to 6.43) | - | ⨁⨁⨁◯ Moderate |  |
|  |  |  |  |  |  |  | - | 0.0% |  | **0 fewer per 1,000** (from 0 fewer to 0 fewer) |  |  |
| **Angle- no meta - Knee peak flexion angle in pre-swing (°)** | | | | | | | | | | | | |
| 1 | non-randomised studies | not serious | not serious | not serious | serious | all plausible residual confounding would reduce the demonstrated effect dose response gradient | 20 cases 12 controls | | **RR 0.40** (-3.75 to 4.55) | - | ⨁⨁⨁◯ Moderate |  |
|  |  |  |  |  |  |  | - | 0.0% |  | **0 fewer per 1,000** (from 0 fewer to 0 fewer) |  |  |
| **Angle- no meta - Ankle peak dorsiflexion angle in single support (°)** | | | | | | | | | | | | |
| 1 | non-randomised studies | not serious | not serious | not serious | serious | strong association all plausible residual confounding would reduce the demonstrated effect dose response gradient | 20 cases 12 controls | | **RR 2.90** (0.78 to 5.02) | - | ⨁⨁⨁⨁ High |  |
|  |  |  |  |  |  |  | - | 0.0% |  | **0 fewer per 1,000** (from 0 fewer to 0 fewer) |  |  |
| **Angle- no meta - Ankle peak dorsiflexion angle in pre-swing (°)** | | | | | | | | | | | | |
| 1 | non-randomised studies | not serious | not serious | not serious | serious | very strong association all plausible residual confounding would reduce the demonstrated effect dose response gradient | 121 cases 156 controls | | **RR 4.70** (2.72 to 6.68) | - | ⨁⨁⨁⨁ High |  |
|  |  |  |  |  |  |  | - | 0.0% |  | **0 fewer per 1,000** (from 0 fewer to 0 fewer) |  |  |
| **Angle2 - Hip sagital ROM (°)** | | | | | | | | | | | | |
| 2 | non-randomised studies | not serious | serious | not serious | not serious | strong association all plausible residual confounding would reduce the demonstrated effect dose response gradient | 91 cases 120 controls | | **RR -4.58** (-7.58 to -1.58) | - | ⨁⨁⨁⨁ High |  |
|  |  |  |  |  |  |  | - | 0.0% |  | **0 fewer per 1,000** (from 0 fewer to 0 fewer) |  |  |
| **Angle2 - Hip frontal ROM (°)** | | | | | | | | | | | | |
| 1 | non-randomised studies | not serious | not serious | not serious | not serious | strong association all plausible residual confounding would reduce the demonstrated effect dose response gradient | 91 cases 120 controls | | **RR -2.25** (-3.32 to -1.18) | - | ⨁⨁⨁⨁ High |  |
|  |  |  |  |  |  |  | - | 0.0% |  | **0 fewer per 1,000** (from 0 fewer to 0 fewer) |  |  |
| **Angle2 - Hip mean rotation (°)** | | | | | | | | | | | | |
| 1 | non-randomised studies | not serious | not serious | not serious | not serious | strong association all plausible residual confounding would reduce the demonstrated effect dose response gradient | 91 cases 120 controls | | **RR 1.72** (-8.08 to 11.52) | - | ⨁⨁⨁⨁ High |  |
|  |  |  |  |  |  |  | - | 0.0% |  | **0 fewer per 1,000** (from 0 fewer to 0 fewer) |  |  |
| **Angle2 - Hip mean sagittal ROM (°)** | | | | | | | | | | | | |
| 1 | non-randomised studies | not serious | not serious | not serious | not serious | all plausible residual confounding would reduce the demonstrated effect dose response gradient | 91 cases 120 controls | | **RR -3.36** (-6.98 to 0.26) | - | ⨁⨁⨁⨁ High |  |
|  |  |  |  |  |  |  | - | 0.0% |  | **0 fewer per 1,000** (from 0 fewer to 0 fewer) |  |  |
| **Angle2 - Hip mean frontal ROM (°)** | | | | | | | | | | | | |
| 1 | non-randomised studies | not serious | not serious | not serious | not serious | all plausible residual confounding would reduce the demonstrated effect dose response gradient | 121 cases 156 controls | | **RR 0.38** (-0.75 to 1.51) | - | ⨁⨁⨁⨁ High |  |
|  |  |  |  |  |  |  | - | 0.0% |  | **0 fewer per 1,000** (from 0 fewer to 0 fewer) |  |  |
| **Angle2 - Knee sagital ROM (°)** | | | | | | | | | | | | |
| 2 | non-randomised studies | not serious | not serious | not serious | not serious | strong association all plausible residual confounding would reduce the demonstrated effect dose response gradient | 91 cases 120 controls | | **RR -6.35** (-8.99 to -3.71) | - | ⨁⨁⨁⨁ High |  |
|  |  |  |  |  |  |  | - | 0.0% |  | **0 fewer per 1,000** (from 0 fewer to 0 fewer) |  |  |
| **Angle2 - Knee mean flexion/ extention** | | | | | | | | | | | | |
| 1 | non-randomised studies | not serious | not serious | not serious | not serious | all plausible residual confounding would reduce the demonstrated effect dose response gradient | 121 cases 156 controls | | **RR -9.75** (-28.86 to 9.36) | - | ⨁⨁⨁⨁ High |  |
|  |  |  |  |  |  |  | - | 0.0% |  | **0 fewer per 1,000** (from 0 fewer to 0 fewer) |  |  |
| **Angle2 - Ankle sagital ROM (°)** | | | | | | | | | | | | |
| 2 | non-randomised studies | not serious | not serious | not serious | not serious | all plausible residual confounding would reduce the demonstrated effect dose response gradient | 91 cases 120 controls | | **RR -0.88** (-2.62 to 0.86) | - | ⨁⨁⨁⨁ High |  |
|  |  |  |  |  |  |  | - | 0.0% |  | **0 fewer per 1,000** (from 0 fewer to 0 fewer) |  |  |
| **Angle2 - Ankle mean sagittal ROM (°)** | | | | | | | | | | | | |
| 1 | non-randomised studies | not serious | not serious | not serious | not serious | all plausible residual confounding would reduce the demonstrated effect dose response gradient | 121 cases 156 controls | | **RR 0.42** (-0.92 to 1.77) | - | ⨁⨁⨁⨁ High |  |
|  |  |  |  |  |  |  | - | 0.0% |  | **0 fewer per 1,000** (from 0 fewer to 0 fewer) |  |  |
| **Standard sagittal - Cadence(steps/s)** | | | | | | | | | | | | |
| 4 | non-randomised studies | not serious | serious | not serious | serious | all plausible residual confounding would reduce the demonstrated effect dose response gradient | 77 cases 86 controls | | **RR -0.40** (-0.92 to 0.11) | - | ⨁⨁◯◯ Low |  |
|  |  |  |  |  |  |  | - | 0.0% |  | **0 fewer per 1,000** (from 0 fewer to 0 fewer) |  |  |
| **Standard sagittal - Stride Length (m)** | | | | | | | | | | | | |
| 3 | non-randomised studies | not serious | very serious | not serious | serious | strong association all plausible residual confounding would reduce the demonstrated effect dose response gradient | 71 cases 71 controls | | **RR -1.32** (-2.43 to -0.21) | - | ⨁⨁◯◯ Low |  |
|  |  |  |  |  |  |  | - | 0.0% |  | **0 fewer per 1,000** (from 0 fewer to 0 fewer) |  |  |
| **Standard sagittal - Velocity (m/s)** | | | | | | | | | | | | |
| 5 | non-randomised studies | not serious | serious | not serious | serious | very strong association all plausible residual confounding would reduce the demonstrated effect dose response gradient | 97 cases 98 controls | | **RR -1.22** (-1.78 to -0.65) | - | ⨁⨁⨁⨁ High |  |
|  |  |  |  |  |  |  | - | 0.0% |  | **0 fewer per 1,000** (from 0 fewer to 0 fewer) |  |  |
| **Standard sagittal - Double support (s)** | | | | | | | | | | | | |
| 4 | non-randomised studies | not serious | very serious | not serious | serious | strong association all plausible residual confounding would reduce the demonstrated effect dose response gradient | 99 cases 99 controls | | **RR 1.12** (0.19 to 2.04) | - | ⨁⨁◯◯ Low |  |
|  |  |  |  |  |  |  | - | 0.0% |  | **0 fewer per 1,000** (from 0 fewer to 0 fewer) |  |  |

**CI:** confidence interval; **RR:** risk ratio
